# fMRI Neurofeedback Training for Increasing Anterior Cingulate Cortex Activation in Adult Attention Deficit Hyperactivity Disorder. An Exploratory Randomized, Single-Blinded Study

**DOI:** 10.1371/journal.pone.0170795

**Published:** 2017-01-26

**Authors:** Anna Zilverstand, Bettina Sorger, Dorine Slaats-Willemse, Cornelis C. Kan, Rainer Goebel, Jan K. Buitelaar

**Affiliations:** 1 Department of Cognitive Neuroscience, Maastricht University, Maastricht, The Netherlands; 2 Department of Psychiatry, Icahn School of Medicine at Mount Sinai, New York, New York, United States of America; 3 Karakter University Centre for Child and Adolescent Psychiatry, Nijmegen, The Netherlands; 4 Department of Psychiatry, Donders Institute for Brain, Cognition and Behaviour, Radboud University Nijmegen Medical Centre, Nijmegen, The Netherlands; 5 Department of Psychiatry, Radboud University Medical Center, Nijmegen, The Netherlands; 6 Department of Neuroimaging and Neuromodeling, Netherlands Institute for Neuroscience, Amsterdam, The Netherlands; 7 Department of Cognitive Neuroscience, Donders Institute for Brain, Cognition and Behaviour, Radboud University Nijmegen Medical Centre, Nijmegen, The Netherlands; National University of Defense Technology College of Mechatronic Engineering and Automation, CHINA

## Abstract

Attention Deficit Hyperactivity Disorder (ADHD) is characterized by poor cognitive control/attention and hypofunctioning of the dorsal anterior cingulate cortex (dACC). In the current study, we investigated for the first time whether real-time fMRI neurofeedback (rt-fMRI) training targeted at increasing activation levels within dACC in adults with ADHD leads to a reduction of clinical symptoms and improved cognitive functioning. An exploratory randomized controlled treatment study with blinding of the participants was conducted. Participants with ADHD (n = 7 in the neurofeedback group, and n = 6 in the control group) attended four weekly MRI training sessions (60-min training time/session), during which they performed a mental calculation task at varying levels of difficulty, in order to learn how to up-regulate dACC activation. Only neurofeedback participants received continuous feedback information on actual brain activation levels within dACC. Before and after the training, ADHD symptoms and relevant cognitive functioning was assessed. Results showed that both groups achieved a significant increase in dACC activation levels over sessions. While there was no significant difference between the neurofeedback and control group in clinical outcome, neurofeedback participants showed stronger improvement on cognitive functioning. The current study demonstrates the general feasibility of the suggested rt-fMRI neurofeedback training approach as a potential novel treatment option for ADHD patients. Due to the study’s small sample size, potential clinical benefits need to be further investigated in future studies.

Trial Registration: ISRCTN12390961

## Introduction

Attention Deficit Hyperactivity Disorder (ADHD) is a childhood-onset neuropsychiatric disorder characterized by a pervasive pattern of inattention, and/or hyperactivity and impulsivity [1,2]. The disorder persists into adulthood in one third of the cases or more, with prevalence in adults being 2–4% [3–5]. The first-line treatment is prescription of medication, mostly psychostimulants. However, response rates in adults are only 20–50% [6], evidence for long-term efficacy of medication is inconsistent [7] and there are concerns about potential side-effects of long-term use of medication [8]. Consequently, novel non-pharmacological treatments are currently being developed, among which electroencephalography (EEG) neurofeedback. Neurofeedback training aims at the remediation of aberrant neuronal functioning, by allowing participants to gain self-control over certain brain-signal aspects, such as theta/beta frequency ratio in EEG neurofeedback training.

A systematic and comprehensive meta-analysis of non-pharmacological treatments for ADHD documented significant treatment effects for EEG neurofeedback, but effects were substantially attenuated when the assessment of outcome was based on blinded raters [9]. A recent double-blind randomized placebo-controlled EEG-neurofeedback study in children and adolescents with ADHD was unable to establish positive treatment effects on clinical symptoms and neurocognitive performance after frequency neurofeedback was compared to placebo-neurofeedback [10,11]. Consequently, this has spurred interest into the development of alternative neurofeedback methods, as for example neurofeedback based on real-time functional magnetic resonance imaging (rt-fMRI), which may be advantageous due to its higher spatial resolution and full brain coverage when compared to EEG. Current state of the art real-time processing techniques allow using fMRI signal for guided self-regulation of brain activation aimed at normalization of deviant brain activation patterns [12]. Importantly, participants are able to control specific aspects of their brain activation patterns, leading to specific changes in behavior [12,13]. For example, up-regulation of activation levels in the motor network has been shown to lead to shorter reaction times in a motor task [14], while up-regulation of the speech network improved accuracy in a language task ([15], for review see [16]). Further, exploratory investigations have indicated a benefit of rt-fMRI guided up- or down-regulation in clinical populations with chronic pain, tinnitus, Parkinson’s disease, stroke, mood and anxiety disorders [17–23]. However, the efficacy of rt-fMRI neurofeedback training in ADHD has not been investigated so far.

The current study was designed to target impaired cognitive control and attention in adults with ADHD by neurofeedback guided self-regulation of dorsal anterior cingulate cortex (dACC). Impaired cognitive control and attention are the most consistently found abnormalities in this clinical population, and are associated with deviant functioning of frontal, cingulate and parietal cortical brain regions [24]. The dACC is the brain region that has been most often linked to core ADHD symptoms [24]. Neuroimaging research using fMRI demonstrates hypo-activation of dACC in patients with ADHD, compared to non-ADHD individuals, specifically during tasks that require effortful control, e.g., interference tasks, continuous performance tests, switch tasks, and response inhibition tasks [24–29]. Moreover, hypo-activation of the dACC was found to normalize after successful treatment with ADHD medication [26], suggesting that normalization of dACC activity is a crucial component of a successful treatment.

The aim of the current study was to train individuals with ADHD to voluntarily up-regulate activation levels in the dACC through rt-fMRI neurofeedback training. We conducted an exploratory randomized controlled treatment study with blinding of the participants to investigate first, if self-regulation of dACC activation level could be achieved, and second, if rt-fMRI neurofeedback training would reduce ADHD symptoms and improve cognitive functioning. Participants attended four weekly rt-fMRI neurofeedback training sessions (60-min training time/session). We assessed ADHD symptoms and cognitive functioning at baseline, a week prior to the training, and a week after training. Participants in the control group underwent the same procedure, but were not provided with neurofeedback information.

## Methods

### Participants

Participants were recruited among referrals to the Department of Psychiatry of the Radboud University Medical Center (Nijmegen, The Netherlands). Participants were included if: they were diagnosed with ADHD according to the DSM-IV TR criteria (American Psychiatric Association, 2000); were older than 18 years; psychotropic drug-naive or -free, or being on a fixed dose of ADHD medication (psychostimulant, atomoxetine or bupropion) for the study period; passed fMRI screening criteria; and had an IQ > 90 according to Block Design and Vocabulary test of the Wechsler Adult Intelligence Scale (WAIS-IV-NL, [30,31]). The administered short version (Vocabulary, Block design) was selected due to its high validity coefficient (0.85) relative to the full intelligence test [32]. Participants were excluded if: they participated in another clinical trial simultaneously; participated previously in neurofeedback training; had another significant medical condition or regular use of medication other than ADHD medication; current diagnosis of one or more Axis-I diagnosis other than ADHD according to the DSM-IV TR criteria (American Psychiatric Association, 2000) (e.g., depression, psychosis, tics, autism, eating disorder); current alcohol or drug abuse according to the DSM-IV TR criteria (American Psychiatric Association, 2000). The recruitment for our study started on May 1^st^, 2013 and the last follow-up was concluded on June 30^th^, 2014.

The presence of ADHD symptoms in childhood and (current) adulthood was assessed using a Semi-Structured Interview for ADHD [33] (see http://www.divacenter.eu/). This interview has been used in previous studies of adult ADHD and shown to be both reliable and valid [4,33–35]. Confirmation of the developmental history and childhood occurrence of ADHD symptoms was obtained from the parents or, when unavailable, an older sibling of the patient. In addition, the Dutch version of the ADHD-DSM-IV Rating Scale [36] was completed by patient, spouse, parent, and investigator to gather information on the exact DSM-IV criteria for ADHD in childhood and adulthood. The following was required for assignment of a full diagnosis of adult ADHD: (1) at least six of the nine DSM-IV criteria for inattention and/or hyperactivity/impulsivity had to be met for diagnosis of childhood ADHD and at least five of the nine criteria for diagnosis of adult ADHD; (2) a chronic course of persistent ADHD symptoms from childhood to adulthood had to be reported; and (3) a moderate to severe level of impairment that can be attributed to the symptoms of ADHD had to be experienced. The cut-off point of five out of the nine criteria for diagnosis of adult ADHD is based upon the literature and epidemiological data using the same DSM-IV ADHD Rating Scale [4,37] and consistent with the DSM-5 algorithm for ADHD.

**Fig 1:**
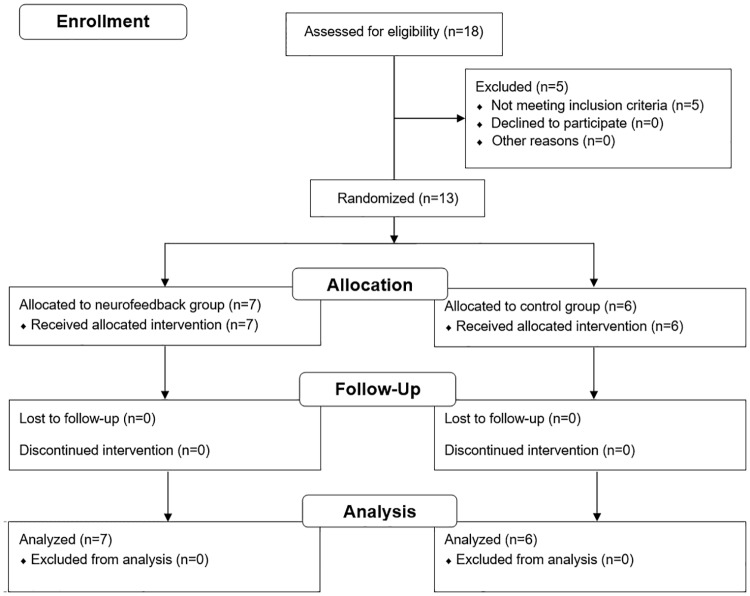
CONSORT Flow Diagram. Eighteen participants volunteered and were screened, thirteen participants were included in the study and randomly assigned to a group.

Eighteen participants volunteered and were screened for inclusion and exclusion criteria. Five participants had to be excluded because they did not fulfill the IQ criterion (Fig 1, S1 File). Thirteen participants were enrolled in the study (Table 1). Participants were randomly assigned to a group using a minimization procedure (sequential balancing) with the factors IQ score, ADHD medication, and the DSM-IV rating scale scores for ADHD symptoms [4]. This restricted randomization procedure has been shown to be efficient in balancing several factors in studies with a small sample size [38,39]. The allocation was performed by implementing a computerized minimization algorithm as described in Borm and colleagues (2005) [39]. Sample size calculations were performed based on the expected training effect using a repeated measures design. All participants received a small financial compensation (8 €/hour), and gave their written informed consent prior to the presented study, which was conducted in conformity with the Declaration of Helsinki and approved by the local Medical Ethics Committee ‘Commissie Mensgebonden Onderzoek Regio Arnhem-Nijmegen’ on September 27^th^, 2012 (S2 File). The study was registered at the ISRCTN registry (http://www.isrctn.com/ISRCTN12390961). This registration was completed after enrolment of participants started, with this delay being due to oversight. The authors confirm that all ongoing and related trials are registered.

**Table 1:** Characteristics of study participants.

Variables (Mean +/- SD) (score pre-testing)	Controls (n = 6)	Neurofeedback (n = 7)	Between-Group Test
Gender (male/female)	3 male/ 3 female	3 male/ 4 female	*p* = 0.78
Age	39.8 (15.0)	34.0 (11.0)	*p* = 0.44
IQ (WAIS VC/BD)	112 (14)	110 (9)	*p* = 0.73
ADHD medication (yes/no)	3 yes/ 3 no	3 yes/ 4 no	*p* = 0.73
ADHD attention (DSM-IV score)	6.3 (1.6)	7.0 (1.2)	*p* = 0.41
ADHD impulsivity/hyperactivity (DSM-IV score)	6.2 (2.7)	6.4 (2.2)	*p* = 0.85
Interference (MSIT, interference delay in ms)	360 (28)	365 (60)	*p* = 0.86
Vigilance (SA-DOTS, z-score)	1.0 (2.2)	0.8 (1.4)	*p* = 0.84
Response inhibition (SA-DOTS, z-score)	1.4 (1.4)	2.8 (3.5)	*p* = 0.36
Response inhibition (SART, % error trials)	36% (24%)	31% (24%)	*p* = 0.71
Visual WM accuracy (2-back, % accuracy)	64% (17%)	67% (19%)	*p* = 0.74
Verbal WM (WAIS DS, IQ score)	104 (17)	96 (9)	*p* = 0.31
Verbal WM (WAIS LNS, IQ score)	109 (11)	99 (7)	*p* = 0.07

During the study period, all participants were either on a stable dose or free of medication for ADHD symptoms (control group: one participant: 100 mg/daily of atomoxetine hydrochloride (Strattera), one participant: 72 mg methylfenidaathydrochloride (Concerta), one participant: 18 mg methylfenidaathydrochloride (Concerta); neurofeedback group: one participant: 30 mg/daily dexamfetamine, one participant: 15 mg/daily dexamfetamine, one participant: 72 mg/daily methylfenidaathydrochloride (Concerta)). *WAIS = Wechsler Adult Intelligence Scale*, *ADHD = Attention Deficit Hyperactivity Disorder*, *VC = Vocabulary*, *BD = Block Design*, *MSIT = Multi Source Interference task*, *SA-DOTS = Sustained Attention DOTS task*, *SART = Sustained Attention to Response Task*, *WM = Working memory*, *DS = Digit Span*, *LNS = Letter Number Sequencing*.

### General procedure

**Fig 2:**
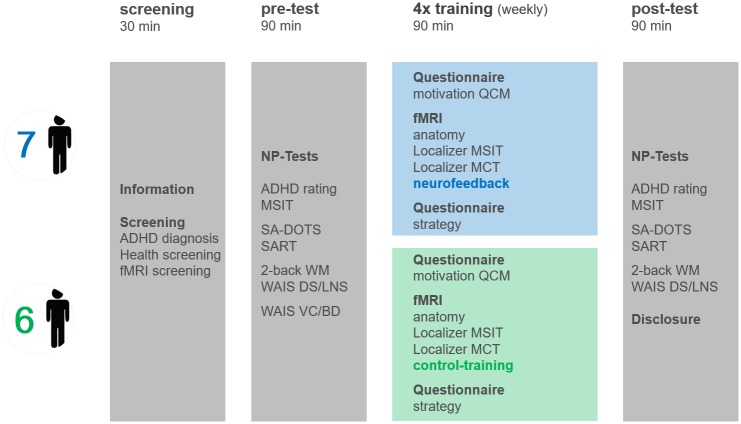
Study design. After enrollment, participants attended six weekly sessions. A week prior to the training, baseline ADHD symptoms and cognitive functioning were assessed (*pre-test*). Then the four weekly training sessions commenced (60-min training time/session). A week after, the last training session, the behavioral post-assessment, was done (*post-test*). *ADHD = Attention Deficit Hyperactivity Disorder*, *fMRI = functional magnetic resonance imaging*, *NP-test = Neuropsychological tests*, *MSIT = Multi Source Interference task*, *SA-DOTS = Sustained Attention DOTS task*, *SART = Sustained Attention to Response Task*, *2-back WM = 2-back Working memory task*, *WAIS = Wechsler Adult Intelligence Scale*, *DS = Digit Span*, *LNS = Letter Number Sequencing*, *VC = Vocabulary*, *BD = Block Design*, *QCM = Questionnaire of Current Motivation*, *MCT = Mental Calculation Task*.

After enrollment, participants attended six weekly sessions. A week prior to the training, baseline ADHD symptoms and cognitive functioning were assessed (*pre-test*). Then the four weekly training sessions commenced, and a week after the last training session, the behavioral post-assessment was done (*post-test*) (Fig 2). All thirteen included participants were able to complete all pre- and post-assessments. One participant in the neurofeedback group and one participant in the control group only participated in three instead of four weekly training sessions, due to technical problems with the MRI scanner. During the 90-min *pre-test*, participants first completed the ADHD DSM-IV rating scale [4], then several neuropsychological tasks to assess cognitive functioning (see description below), and the (short version of the) intelligence test [31]. During the 90-min *post-test*, participants completed the same tasks in the same order, except for the intelligence test. During the first MRI session, all participants, including the control group, were informed that the goal of the study was to investigate if up-regulation of dACC activation levels through performing a mental task would have a positive impact on ADHD symptoms and cognitive functioning. Immediately before the training in the MRI scanner and after the instruction, participants were asked to complete a Questionnaire of Current Motivation to assess their motivational state (QCM, [40]). The QCM measures individual differences in current motivation and expectation of success using four different scales: *perceived challenge*, *level of interest*, *mastery confidence* and *incompetence fear*. The following rt-fMRI neurofeedback training session consisted of a 7-min anatomical scan, two 9-min *localization runs*, used to functionally define the target regions, three 8-min *training runs* performed by both groups, providing feedback for neurofeedback participants only, and an 8-min *transfer run*, during which both groups did not receive feedback.

### Localization of dACC target regions

During the first *localization run* for detecting individual dACC activation, participants performed the *multi-source interference task* (MSIT, [41]), which was specifically designed to functionally localize the dACC as the critical node in prefrontal cognitive control/attention circuits impaired in ADHD [41,42]. This task combines three different tasks to maximally increase cognitive interference: the Eriksen Flanker task, the Counting Stroop, and the Simon effect task [42]. Trials were presented blocked as in the original article, using the same trial parameters [41], but inserting an intermittent rest period of 18–24 s between blocks (total duration: 9 min). The dACC target region was defined functionally during online fMRI analysis for each participant/training session by contrasting activation during interference blocks (*interference*) with intermittent rest periods of rt-fMRI data.

In a second dACC *localization run*, participants were instructed to perform a *mental calculation task* (MCT), similar to the mental task they performed during later *training runs*. This task has been shown to activate dACC across different task variants [43,44]. During *mental calculation* blocks, participants were asked to start with the number 100 and keep subtracting a single digit number, which was selected individually such that the task was of medium difficulty. During the control condition, participants were asked to mentally rehearse a self-selected song (*mental singing task*), which was easy and well-known to them. This localization run included five 26-s blocks of each condition with intermittent 26-s resting periods (total duration: 9 min). The second dACC localization task was employed to ensure the definition of the target regions on an individual level in all sessions, to verify functional overlap between the MSIT and *mental calculation* task post-hoc, and as warm-up task prior to the neurofeedback training.

### Training procedure

Prior to scanning, all participants were told that the rationale of the mental training was to train their attention. They were instructed how to vary the difficulty of the *mental calculation* task by systematically changing three different task aspects: 1) tempo, 2) magnitude of the numbers, and 3) variations in the operation rule and asked to practice the task out aloud under supervision of the experimenter. All instructions for all tasks used in this study were standardized, supported by computerized visual instructions, given by the same experimenter and repeated during each scanning session. The specific instructions for the training were developed based on fMRI studies on activation levels during different arithmetic operations [43,44], and participants were reminded of these instructions in the scanner at the beginning of each *training run*. Participants of both groups were told that the specific task would be cued by a red box in a visual thermometer display, indicating either: rest (no cue), *medium task difficulty* (cue at medium height) or *high task difficulty* (cue at top, S1 Fig). Both groups were asked to adapt the task throughout the experiment as necessary, in order to maintain an individual medium and high difficulty level. Participants in the control group were asked to adapt the task difficulty levels based on insight, the visual display only served as a cue in this group, as they were not aware of participating in a neurofeedback study. In the neurofeedback group, the thermometer display showed the actual activation level of the dACC target region (S1 Fig). Participants in the neurofeedback group were instructed to adapt the mental calculation performance/difficulty level of the task in order to reach the indicated brain activation level (medium and high) based on the provided neurofeedback information. Finally, neurofeedback group participants were demonstrated the common noise level of the fMRI signal and the delay of the blood-oxygen-level dependent (BOLD) response through a 10-min simulation program. Both groups were instructed to minimize motion in the scanner and informed that we would be watching their brain activation levels during the training through rt-fMRI data analysis.

Each *training run* consisted of eight 30-s task blocks (four *medium/*four *high level*) in pseudo-randomized order, with intermittent 20-s rest periods (total duration: 8 min). All participants performed three *training runs* per session, followed by an 8-min *transfer run* during which no feedback was provided in either group, in order to test knowledge transfer.

### MRI imaging parameters

The MRI images were acquired at the Donders Centre for Cognitive Neuroimaging, Radboud University Nijmegen, on a 3T scanner (Tim Trio, Siemens Healthcare, Germany), equipped with a 32-channel head coil. Functional images were acquired with a repeated single-shot echo-planar imaging (EPI) sequence with TE = 30ms, TR = 2000ms, FA = 80°, FOV = 192x192mm^2^, matrix = 64x64, voxel size 3x3x3 mm^3^, bandwidth = 1628Hz/Px, 35 slices per volume with whole-brain coverage. Anatomical images were collected with a 3D MPRAGE sequence: TR = 2300 ms, TE = 3.92 ms, FOV = 256x256mm^2^, voxel size 1x1x1 mm^3^, 192 slices.

### Real-time MRI data analysis

#### Pre-processing

Anatomical images were processed using BrainVoyager QX (Version 2.7, Brain Innovation, Maastricht, The Netherlands), and loaded into Turbo-BrainVoyager (Version 3.2, Brain Innovation, Maastricht, The Netherlands) for rt-MRI data analysis. After discarding the first four volumes of each functional run, functional and anatomical data were automatically aligned. Functional data was pre-processed in real-time using intra-session 3D rigid-body motion correction and linear drift confound predictors. An online voxel-wise general linear model (GLM) was computed, convolving the task predictors with a standard two-gamma hemodynamic response function. The data from the *localization runs* was additionally high-pass filtered with a GLM Fourier basis set (3 cycles/run), and thresholded at t = 3 prior to the definition of the dACC target regions, with an additional cluster threshold of four significant voxels.

#### Localization of dACC target regions

The individual dACC target regions were defined based on the first localization task (MSIT), contrasting the *interference* with the *rest* condition, as pilot measurements had shown that this contrast was more robust than contrasting *interference* versus *no interference*, while localizing the same network. If no significant cluster within dACC could be ascertained using the first localization task’s data (ca. 10% sessions), the dACC target regions were defined based on the data of the second localization task (MCT), contrasting *mental calculation* with *rest*. Generally, the most anterior dACC cluster was selected, as the anterior dACC showed the strongest under-activation in ADHD patients [26].

#### Generation of neurofeedback information

For the neurofeedback, the percent signal change (PSC) in the dACC target region was computed relative to a 14-s baseline from the previous *rest* period, being updated after each acquired imaging volume (every two seconds). The maximum PSC of the thermometer display was adjusted individually per session to be 150% of the mean activation level in the dACC target region during the *mental calculation* condition (if <0.3% PSC, the activation level during the *interference* condition of the MSIT was used as a reference). The maximal PSC displayed in the neurofeedback group increased slightly over sessions (session 1: 0.81%, session 2: 0.86%, session 3: 0.90%, session 4: 0.95%).

### Post-hoc analysis of fMRI data

#### Preprocessing

For post-hoc fMRI data analysis, the same preprocessing parameters as during real-time data analysis were applied. Additionally, data was spatially normalized to Talairach space [45] and functional runs during which participants moved more than 5 mm/degree in any direction/rotation were excluded (one neurofeedback participant: fourth session *training runs* 2 and 3, *transfer run*; one control participant: second session *training runs* 1 and 2, third session *training run* 3, fourth session *training run* 2). FMRI data quality was evaluated by computing *mean displacement* (average motion from volume to volume) and *temporal signal-to-noise ratio* (tSNR), averaged across voxels within individual dACC target regions after removal of task activation by regression [46].

#### Evaluation of dACC localization procedure

To verify the online selection of the dACC target regions, we conducted a whole-brain random-effects GLM analysis, thresholding maps using an initial voxel-threshold of α = 0.05 [47] and correcting for multiple comparisons using cluster-size thresholding with a cluster-level false positive rate of α = 0.05 [47,48]. The contrasts of interest were *interference vs*. *no interference* and *mental calculation vs*. *mental singing*. To further evaluate task performance across groups, a region-of-interest analysis of the BOLD response in the dACC target regions was performed. The estimated beta weights of the average BOLD response were extracted, and analyzed in SPSS Statistics (IBM SPSS Statistics 21; IBM Corporation, Armonk, NY, USA). They were submitted to statistical analysis using repeated measures GLM with linear contrasts, modeling the factors *task* (*interference*, *no interference; mental calculation*, *mental singing)* and *time (session)*. Effect sizes were estimated using partial eta squared (η_p_), which describes the proportion of the total variability in the dependent variable attributable to an effect [49].

#### Evaluation of training performance

To evaluate self-regulation performance during *training* and *transfer runs*, the estimated beta weights of the dACC target regions for these runs were extracted and submitted to a repeated measures GLM with linear contrasts, modeling the factors *task* (*50% difficulty*, *100% difficulty*), *time* (*run*, *session*) and *group (neurofeedback*, *no feedback)*. To further evaluate the nature of learning effects, additional planned comparisons were conducted to evaluate at which point of the training activation levels increased (comparisons: session one vs. two, session two vs. three and session three vs. four). Bonferroni correction was applied to correct for multiple comparisons. Finally, questionnaire data (QCM) was analyzed using the factors *time* (*session*) and *group (neurofeedback*, *no feedback)*.

#### Exploration of performance-predicting factors

To explore which factors may predict successful performance in neurofeedback training, an exploratory analysis was conducted investigating if baseline cognitive functioning would predict performance during self-regulation. Three performance indices were calculated based on the extracted beta weights from the dACC target regions. First, an index of *general task performance* (mean activation level across all sessions and activation-level conditions), second, an index of *improvement over sessions* (increase in activation level over sessions) and third, an index of *improvement in differential modulation* (increase in differential activation between *50% vs*. *100% difficulty* over consecutive sessions, S2 Fig). Correlations between performance indices and cognitive performance during *pre-test* neuropsychological were computed.

### Pre-post behavioral assessment

Individuals with ADHD are found to exhibit significant impairments with medium effect sizes on a range of executive functioning tasks. The strongest and most consistent performance deviations are found for sustained attention tasks requiring response inhibition or vigilance, as well as working memory tasks, particularly when spatial working memory is required [50,51]. We employed several tasks shown to be sensitive measures of neuropsychological functioning in ADHD patients. All computerized tasks used were programmed and presented using Presentation software package (Version 16, Neurobehavioral Systems Inc., Albany, CA, USA).

The first continuous performance tasks employed was the Sustained Attention Dots task (SA-DOTS, [52]). The SA-DOTS is a computerized visual sustained attention task, during which 50 series of twelve dot patterns are presented randomly, a total of 600 dot patterns (15 min). Participants are required to press “yes” when a four-dot patterns are presented (target, 1/3 of trials), and “no” for three/five-dot patterns (non-targets, 2/3 of trials). Missed targets are considered an index of failed response inhibition, while false alarms are assumed to reflect vigilance [53,54]. The SA-DOTS has an excellent test-retest reliability (0.90–0.94), and provides performance z-scores in reference to a normed age sample [55]. It has also been shown to discriminate ADHD patients from healthy controls [53,54].

The second continuous performance task administered was the computerized Sustained Attention to Response Task (SART, [56]). During 216 trials, one single digit numbers (1–9) is presented each trial (6 min). Participants are instructed to press a button after each digit, withholding their response only when a ‘3’ is presented (non-target, 11% of trials). Due to the high number of targets (89% of trials), a strong response bias towards pressing the button is induced, making this task sensitive for detecting impairments of response disinhibition, which are indexed by the number of false alarms. The task has been shown to be sensitive to discriminating ADHD patients from healthy controls [57,58].

To assess visual working memory, a computerized 2-back visuospatial task was employed (2-back WM, [59]). During each trial, a white square light up at one of nine possible locations on the computer screen. Participants are asked to press a button when the square appears at the same location as two trials ago (25% of trials). In total 10 sequences of 15 trials are presented (9 min). The main outcome measure for indexing working memory performance is the proportion of correct trials, which is calculated by subtracting the proportion of misses and false alarms from the total number of trials. This task has shown to be sensitive to working memory impairments in ADHD patients [59].

For assessment of verbal working memory, two subtests from the standardized WAIS-IV-NL, the Digit Span and Letter-Number Sequencing task, were used. Both were administered verbally, following standard procedures [31]. For both subtests age-referenced norm scores were calculated. The Digit Span has shown to be discriminative in detecting working memory impairments in ADHD [50], has a very good internal consistency (>0.85), and an adequate test-retest reliability (>0.75) [60].

To monitor changes due to repeated training of the first dACC localization task, the MSIT, this task was also performed during pre- and post-testing. We analyzed *interference* delay, the slow-down in reaction time during *interference* trials relative to *no interference* trials, as a measure of capacity to deal with cognitive interference [42].

The MSIT *interference delay*, pre- and post-scores on the ADHD attention and impulsivity scale and all behavioral *pre*- and *post-test* scores were analyzed in SPSS Statistics, using *change* (*pre-test*, *post-test*) and *group (neurofeedback*, *no feedback)* as a factor.

## Results

### Participants

At baseline, participants reported on average of seven out of nine ADHD attention symptoms and six out of nine ADHD hyperactivity/impulsivity symptoms, and slightly above average IQ (Table 1). Three participants in the control group and three participants in the neurofeedback group were on a stable doses of medication (Table 1). During *pre-test*, participants performed below standards of healthy control groups. They responded 17% slower on the cognitive interference task (MSIT [42]), performed 0.9 SD below the norm on vigilance and 2.1 SD below the norm on response inhibition (SA-DOTS [52]), demonstrated a marked increase of false alarms (34% vs. 12% in healthy) during the second response inhibition task (SART [58]), and marked decrease in accuracy (66% vs. 78% in healthy) in the visual working memory task (WM 2-back, [59]) (Table 1).

### Localization of dACC target regions

**Fig 3:**
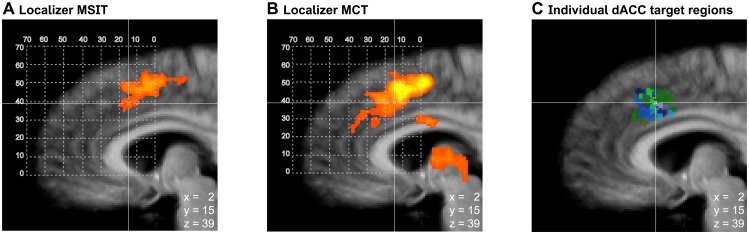
Localization of dACC target regions. Activation during the localization tasks is depicted for the interference task (MSIT, panel A, peak activation x = 8, y = 10, z = 45, Talairach space) and the mental calculation task (MCT, panel B, peak activation x = -8, y = 10, z = 48, Talairach space). To verify if the targeted dACC region was localized/defined within the online procedure, a whole-brain random effects GLM analysis (p<0.05, corrected at cluster level) was performed for the contrast *interference* vs. *no interference* (MSIT, panel A), and the contrast *mental calculation* vs. *mental singing* (MCT, panel B). Panel C depicts the location of the online defined individual dACC target regions around their average location (x = 2, y = 15, z = 39, Talairach space, neurofeedback group = blue tints, control group = green tints). The slightly more anterior location of the dACC target regions respective to the peak of activation may have resulted from a preference for the most anterior clusters during the localization procedure, which have been shown to be hypo-activated in ADHD patients [26].

The post-hoc analysis of the two localization tasks showed that both localizer tasks, the MSIT and the MCT, activated the dACC (Fig 3A and 3B). Both localizers had a similar activation focus, activating overlapping voxels within the dACC (Fig 3A and 3B). The individual dACC target regions defined online coincided well with the region activated during the localization tasks (Fig 3A–3C). The coordinates of the dACC target regions were similar across groups and sessions (S1 Table), and the coordinates were similar as shown by previous studies with ADHD patients [26].

**Fig 4:**
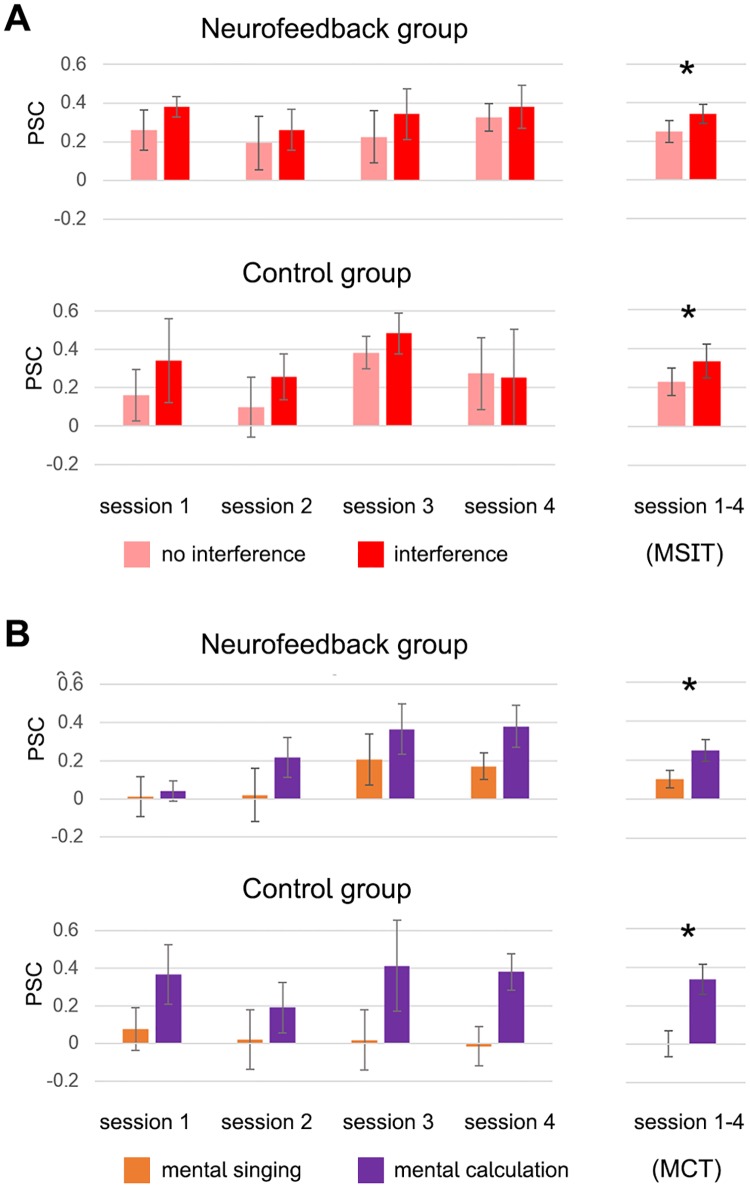
Activation levels within dACC target regions during localization tasks. Participants across groups showed significantly increased activation levels in the dACC target region during the *interference* condition of the MSIT (panel A, marked with an asterisk), and the *mental calculation* condition of the MCT (panel B). There were no changes in performance over time, confirming that both groups consistently performed the task as instructed.

The region-of-interest analysis evaluating performance during *localization runs* revealed that across groups participants showed significantly higher activation in the dACC target regions during the *interference* vs. *no interference* condition (F(1,11) = 19.6, p < 0.001, η_p_^2^ = 0.64, Fig 4) and the *mental calculation* vs. *mental singing* condition (F(1,11) = 9.2, p < 0.01, η_p_^2^ = 0.45, Fig 4). Further, there were no significant changes in activation levels across sessions (linear change over sessions: MSIT: F(1,11) = 0.24, p = 0.63; MCT: F(1,11) = 1.6, p = 0.23, Fig 4) and no significant group differences during performance of either task used during the localization procedure (MSIT: F(1,11) = 0.02, p = 0.90; MCT: F(1,11) = 0.005, p = 0.94). In summary, both groups consistently performed the two *localization tasks* as instructed without any indication of a change in performance over time.

### Training data

**Fig 5:**
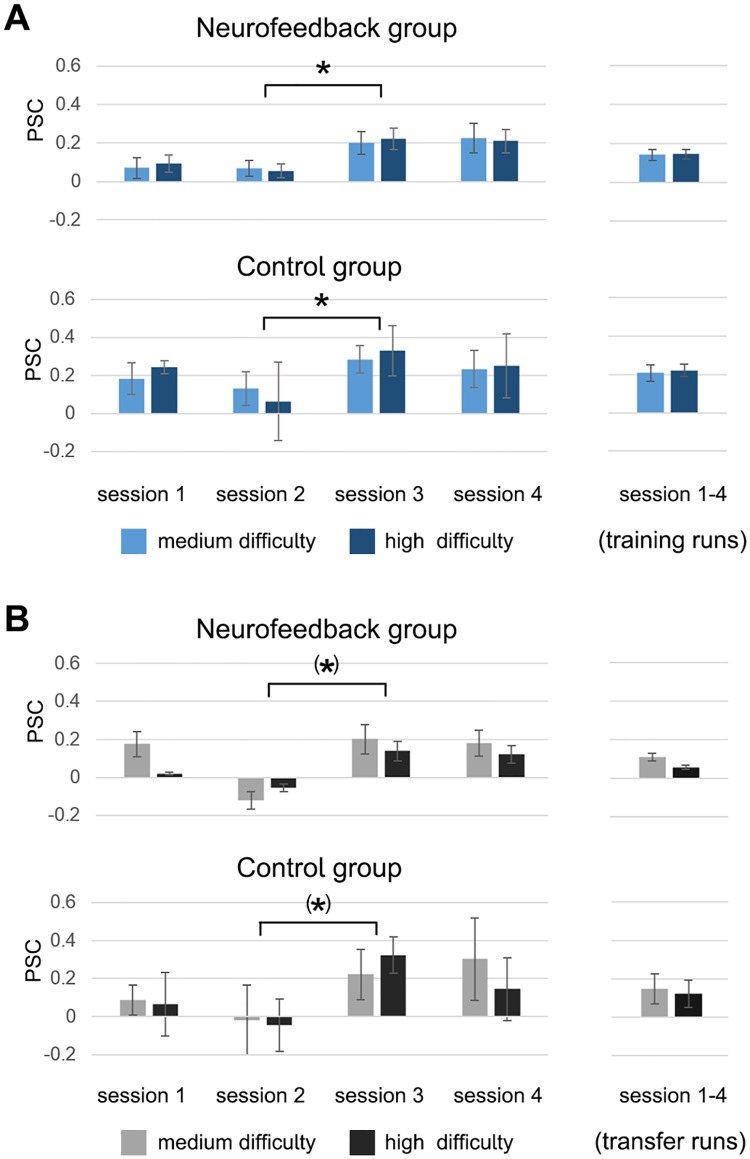
Activation levels within dACC target regions during MRI training and transfer runs. The results demonstrate that all participants achieved the expected up-regulation of activation levels within the dACC target regions with training. Both groups showed a marked and significant increase in dACC activation level from the third session onwards, which was evident during *training runs* (panel A, marked with an asterisk), and marginally significant during *transfer runs* (panel B). Importantly, participants were able to sustain increased activation levels until the end of training. There were no significant group differences and neither group demonstrated ability to differently up-regulate the dACC activation levels during the *medium* and *high difficulty* condition.

The analysis of self-regulation performance during the *training runs* revealed learning effects in both groups. While there was no significant group difference (F(1,11) = 0.8, p = 0.38), both groups showed a marked and significant increase in activation level between the second and the third session, with activation levels remaining high until the end of the training (F(1,11) = 8.4, p < 0.05, η_p_^2^ = 0.43, Fig 5A, S3 File), demonstrating that both groups were able to sustain this increase in activation levels until the end of training. In the neurofeedback group, the observed increase of activation levels from the first to the last session was slightly stronger than in the control group. Further, participants of both groups were not able to differentially up-regulate dACC activation to different target levels (medium and high activation level) during the *50%* and *100% difficulty* condition (F(1,11) = 0.1, p = 0.81). A similar pattern was observed for the *transfer runs*, during which neither group received feedback. There was no significant group difference between neurofeedback and control participants (F(1,11) = 0.3, p = 0.61), but a trend indicated that both groups learned to increase their dACC activation levels from the third training session onwards (F(1,11) = 7.4, p-corrected = 0.06, η_p_^2^ = 0.40, Fig 5B). Again, both groups were not able to differentially up-regulate dACC activation to different target levels (medium and high activation level) (F(1,11) = 0.9, p = 0.36). In summary, both the data from the *training runs*, as well as from the *transfer runs* support the conclusion that learning took place in both groups and that participants were able to maintain upregulation effects until the end of training. The results demonstrate that all participants achieved the expected up-regulation of dACC target region activation levels with training and achieved the same increase during *transfer runs*, when no feedback was provided.

To evaluate which factors may have predicted dACC self-regulation performance, additional analyses regarding MRI data quality, effects of motivation and neuropsychological predictors of performance indices were performed. The exploratory analysis of motion during the *training runs* showed that participants in the neurofeedback group moved significantly less during the training (mean displacement: F(1,10) = 4.8, p = 0.05, η_p_^2^ = 0.33, S3A Fig), even though both groups received the same instruction not to move in the scanner. While fMRI data quality in the neurofeedback group was high (average tSNR = 145), it was significantly reduced in the control group (average tSNR = 98) (F(1,10) = 5.5, p < 0.05, η_p_^2^ = 0.36, S3B Fig). This decrease in fMRI data quality in the control group was linked to individual motion, the two measures were highly correlated within both groups (neurofeedback group: r = -0.77, p<0.001; control group: r = -0.61, p<0.001, S3C Fig). Importantly, neither ability to refrain from motion, nor fMRI data quality changed over time (linear change over sessions: motion F(1,10) = 0.45, p = 0.52; tSNR F(1,10) = 0.89, p = 0.37). However, individual ability to refrain from movement did predict better *general task performance* in the neurofeedback group (r = -0.79, p<0.05, S2 Table).

In contrast to motion parameters, motivation, as measured by the QCM, did change over time. At the end of training, participants perceived the training to be less challenging after (F(1,11) = 12.1, p < 0.01, η_p_^2^ = 0.52) and showed slightly decreased level of interest (F(1,11) = 11.5, p < 0.01, η_p_^2^ = 0.51). Contrary to this, mastery confidence increased with a greater number of sessions (F(1,11) = 5.0, p< 0.05, η_p_^2^ = 0.31). Overall, general motivation scores were high and comparable to levels measured in previous neurofeedback training studies [22]. Importantly, no group differences in motivation emerged over time, with neurofeedback participants perceiving the training as generally more challenging (F(1,11) = 8.5, p < 0.05, η_p_^2^ = 0.44, S4 Fig). Further, individual motivation levels during training did not predict performance during dACC self-regulation (S2 Table).

The exploratory analysis of neuropsychological predictors of the self-regulation performance during dACC revealed that neither IQ, nor ADHD attention or impulsivity score were predictive of performance (S2 Table). However, both better ability to inhibit responses and higher accuracy on working memory tasks predicted larger *improvement over sessions* in the neurofeedback group only (response inhibition false alarms: r = -0.88, p<0.05; WM accuracy r = 0.86, p<0.01, S2 Table). Similarly, better individual scores on response inhibition and working memory predicted larger improvement in differential modulation in the neurofeedback group only (response inhibition % missed: r = -0.91, p<0.01; WM accuracy r = 0.80, p<0.05). On individual level, higher capacity for inhibitory control during self-regulation and better working memory thus lead to improved performance in the neurofeedback group only.

### Pre-post behavioral assessment

Behavioral assessment after four weeks of training revealed that the neurofeedback group improved slightly, but not significantly, on both the ADHD attention and impulsivity score, while the control group improved slightly, but not significantly, regarding impulsivity (Table 2, S4 File). Both groups showed a significant improvement during *post-test* relative to baseline on the cognitive interference task, which was administered both during pre- and post-testing as well as during the dACC localization procedure of each MRI session (MSIT interference delay: F(1,11) = 31.2, p < 0.001, η_p_^2^ = 0.75, see Table 2 for pre-post behavioral assessment). Finally, while differences between groups during *post-test* neuropsychological testing did not reach statistical significance, participants of the neurofeedback group only showed significant improvement on cognitive functioning. During *post-test*, the neurofeedback group, but not control participants, performed significantly better on the sustained visual attention task (SA-DOTS response inhibition: F(1,6) = 5.9, p = 0.05, η_p_^2^ = 0.50, Table 2), improving their performance by 1.7 SD relative to the norm. Second, neurofeedback participants only showed significantly improved accuracy during the visual working memory task at the neuropsychological post-test, improving their accuracy to levels seen in healthy control groups (WM 2-back accuracy: F(1,6) = 7.7, p<0.05, η_p_^2^ = 0.56, Table 2).

**Table 2:** Pre-post behavioral assessment (within group).

Variables assessed during pre-/post-testing	Controls (n = 6) Pre-testing/post-testing, p-value (partial eta^2^)	Neurofeedback (n = 7) Pre-testing/post-testing, p-value (partial eta^2^)
ADHD attention (DSM-IV score)	pre: 6.3 post: 6.7, p = 0.73 (0.03)	pre: 7.0 post: 6.0, p = 0.23 (0.23)
ADHD impulsivity/hyperactivity (DSM-IV score)	pre: 6.2 post: 5.0, p = 0.16 (0.36)	pre: 6.4 post: 5.7, p = 0.31 (0.17)
Interference (MSIT, interference delay ms)	pre: 360 post: 274, p = 0.01 (0.82) *	Pre: 365 post: 290, p = 0.01 (0.68) *
Vigilance (SA-DOTS, false alarms)	pre: 1.0 post: 1.1, p = 0.88 (0.01)	pre: 0.8–0.1, p = 0.10 (0.39)
Response inhibition (SA-DOTS, % missed)	pre: 1.4 post 1.2, p = 0.85 (0.01)	pre: 2.8 post: 1.1, p = 0.05 (0.49) *
Response inhibition (SART, % false alarms)	pre: 36% post: 40%, p = 0.67 (0.04)	pre: 31% post: 31%, p = 1.00 (0.00)
Visual WM accuracy (2-back, % accuracy)	pre: 64% post: 68%, p = 0.46 (0.11)	pre: 67% post: 76%, p = 0.03 (0.56) *
Verbal WM (WAIS DS, IQ score)	pre: 104 post: 111, p = 0.29 (0.22)	pre: 96 post: 101, p = 0.41 (0.12)
Verbal WM (WAIS LNS, IQ score)	pre: 109 post: 104, p = 0.39 (0.15)	pre: 99 post: 100, p = 0.79 (0.01)

Significant effects (p ≤ 0.05) are printed bold and marked with an asterisk. *MSIT = Multi Source Interference task*, *SA-DOTS = Sustained Attention DOTS task*, *SART = Sustained Attention to Response Task*, *WM = Working memory*, *DS = Digit Span*, *LNS = Letter Number Sequencing*.

## Discussion

The goal of this study was to investigate if training individuals with ADHD to voluntarily up-regulate activation levels in the dACC through rt-fMRI neurofeedback training would have beneficial effects on ADHD symptoms and cognitive functioning. In summary, we found that individuals with ADHD were able to up-regulate dACC activation levels through rt-fMRI neurofeedback training and maintain these changes until the end of training in both the neurofeedback and control group. Second, while there was no significant difference between the neurofeedback and control group in clinical outcome, neurofeedback participants showed stronger improvement on neuropsychological functioning.

### Feasibility of the approach

The neurofeedback and control group were well matched on demographic variables, such as gender, age, IQ, and clinical variables, such as ADHD symptoms, medication status and baseline neuropsychological functioning, indicating the usefulness of the selected randomization procedure (minimization) for small sample sizes. Both groups were also representative of the patient population, demonstrating high levels of impairment, both regarding ADHD symptoms and during neuropsychological testing [4,55,58,59,61]. Both groups were highly motivated and demonstrated consistent performance during the localization procedure. The consistent performance on the second localization task, which was similar to the training task, suggests that participants in both groups were generally able to follow the training instruction. Importantly, mastery confidence regarding training increased over sessions, again indicating that participants were able to perform the training task as instructed. The retention rate of the study was 100%, also indicating high general motivation in both groups. The dACC target regions, from which the neurofeedback was derived, could be localized reliably in all subjects and sessions and were located within the targeted network. The analysis on MRI data quality demonstrated that in the neurofeedback group sufficiently high signal quality was achieved for a rt-fMRI experiment [62], as providing continuous neurofeedback during training seemed to attenuate motion during training in this group. Interestingly, performance during neurofeedback training was neither predicted by general IQ, nor by severity of ADHD attention symptom, which makes it feasible to implement training even in more severe cases, as included in this study. Further, exploratory analyses showed that neurofeedback training performance was influenced by capacity for working memory and response inhibition, suggesting that it may be beneficial to perform a cognitive training program prior to neurofeedback or take these factors into consideration during recruitment. The results confirm previous investigations showing that working memory capacity is a predictor of success in EEG neurofeedback training [63,64]. Overall, the presented results demonstrate the general feasibility of the approach as implemented in this study for a neurofeedback training in this patient population.

### Training effects

During self-regulation training, both groups demonstrated similar changes in activation patterns over time. Both groups achieved the expected up-regulation of activation levels in dACC target regions after the second session and were able to maintain this improved capacity for self-regulation until the end of the training, with the neurofeedback group demonstrating a slightly larger increase in activation levels from the beginning until the end of training. Overall, these results indicate that both groups achieved a similar degree of learning. The presented results support that up-regulation of dACC target regions might be achieved through cognitive training alone, as has been suggested in recent research on the influence of working memory training on brain function [65]. However, it remains to be explored in a larger study if there are differential effects of the two type of trainings (*feedback*, *no feedback*). Stronger performance of the neurofeedback group during neuropsychological testing suggests that subtle differences may exist between the groups, supporting that learning in the neurofeedback group may have been more efficient. More generally, the results further confirm previous studies reporting that multiple sessions may be necessary when neurofeedback training is implemented in clinical populations [19,66]. Importantly, from the third session onwards, participants were able to maintain high activation levels even during transfer runs, when no feedback was provided. This further suggests that some generalization of the learned skills took place in the neurofeedback group, enabling them to transfer these skills into a different context. Finally, the presented results show that individuals with ADHD did not achieve graded control of the brain activation level within the dACC as they were not able to differentially modulate the signal to two different levels within four training sessions. This result stands in contrast to previous results in healthy participants, demonstrating that healthy individuals indeed are able to achieve modulation up to at least three brain activation levels [67]. In general, this indicates that the implemented neurofeedback training may have been particularly challenging for individuals with ADHD, and may need further adaptation to the needs of this clinical population.

That the implemented neurofeedback training may have indeed been challenging for this clinical group is further supported by the fact that participants in the neurofeedback group indicated by self-report that they felt significantly more challenged throughout the training, relative to the control group. This stands in contrast to previous neurofeedback trainings in clinical groups, which reported that there was no difference in perceived challenge between the neurofeedback and the non-neurofeedback control group in individuals with anxiety disorder [22]. However, one difference between the current and this previous study is that the current study provided continuous neurofeedback during task performance, while the previous study provided intermittent neurofeedback in-between task blocks [22]. Research systematically comparing these two sorts of neurofeedback in healthy participants (in the motor system), suggests that continuous neurofeedback may be indeed more challenging than intermittent neurofeedback [68]. The reason for this may be that continuous neurofeedback requires participants to monitor the neurofeedback signal while performing a task, which poses a dual-task challenge. Indeed, the mentioned systematic study into different sorts of neurofeedback demonstrated that in some participants activation levels in the neurofeedback region were significantly reduced during continuous neurofeedback, while participants were actually trying to up-regulate the signal [68]. This suggests that participants may actually have to exert more mental effort when up-regulating activation levels guided by continuous neurofeedback as the control group. Moreover, this may be particularly relevant when the aim is to up-regulate activation within the dACC, a region which is known for its involvement in task monitoring [69], and therefore likely to be affected by dual-task demands. In conclusion, participants in the neurofeedback group may thus indeed have had a more challenging task than control participants, when being asked to up-regulate dACC activation levels during continuous neurofeedback. Importantly, however, this did not seem to have an adverse but more likely a beneficial effect on outcome in the neurofeedback group, as neuropsychological functioning was significantly improved in this group only.

### Limitations

The fact that we did not include an additional sham neurofeedback control group, which receives non-valid continuous neurofeedback, may be seen as a limitation of this study. However, the same line of research investigating different sorts of neurofeedback, also demonstrated that sham neurofeedback may be perceived as frustrating and can thus induce a negative performance bias in the control group, limiting the performance of the control group [62,68]. A non-neurofeedback control group with blinding of participants therefore seemed the strictest design choice available. To ensure comparability between the neurofeedback and control group, several measures were taken. First, both groups were instructed in the exact same way, receiving the same information regarding the goal of the study. To both groups, we pointed out that recent neuroscience reports suggested that up-regulation of activation within their individual dACC region by mental effort may be beneficial, and we also stressed that we would monitor their progress by looking at their brain activation levels. That this resulted in high motivation in both groups is supported by the reported levels of interest and mastery confidence, which were high and comparable to previous studies [22]. Second, both groups received an active instruction on how to up-regulate activation levels in dACC region. Also, they were explicitly instructed to keep adapting the task throughout the training, in order to keep themselves challenged. By providing both groups with a very similar and active instruction, we may, unintentionally, have compared two different active interventions, instead of comparing a neurofeedback with a true control training. The instructions given to the control group may be conceptualized as a working memory training, which has been shown to have beneficial effects in ADHD individuals [70,71]. Importantly, however, the present results provide preliminary evidence that the neurofeedback training may have had a stronger beneficial effect than the provided control training, as neuropsychological functioning was improved in the neurofeedback group only.

A second limitation of this study is its limited sample size. Due to the small sample size, any conclusion regarding the potential clinical outcome needs to be drawn carefully. The presented exploratory results confirm the general feasibility of the chosen approach, but cannot be used to evaluate the clinical benefits of rt-fMRI neurofeedback training in adult ADHD individuals. However, the overall positive effects of the neurofeedback training suggest that it is warranted to further explore rt-fMRI neurofeedback training as a novel treatment option in ADHD.

### Future recommendations

The first aim of this study was to establish the feasibility of the suggested approach. The results suggest that the employed recruitment and randomization strategies, standardized task instructions and technical procedures were successfully implemented, as demonstrated by the different indicators for monitoring the study quality. Importantly, the analysis of performance predictors indicated that the approach is suited for ADHD patients with average IQ, even when ADHD symptoms are severe. However, this analysis also indicated that ADHD patients with severe deficits in working memory and inhibitory control profited much less from the training. For future trainings, it may therefore be beneficial to design step-wise trainings to reach a larger group of patients. Rt-fMRI neurofeedback training may need to be complemented with other training modules, aimed at ameliorating the cognitive capacities that were predictive of success, such as working memory and response inhibition. A more elaborate training on motion control prior to scanning may also be useful. Third, an initial training module providing neurofeedback from brain regions that are more easily controllable (e.g., motor system), may be beneficial to accustom participants to the dual-task demands of monitoring continuous neurofeedback during training. Finally, the goal of achieving graded control over the signal may only be attainable with more additional training sessions. Overall, the presented results suggest that the benefits of rt-fMRI neurofeedback training effects may be maximized by additional instruction and practice modules prior to the neurofeedback training itself, therefore supporting a multifaceted interventional approach.

## Conclusions

In conclusion, the presented results suggest that rt-fMRI neurofeedback training may constitute a potential novel treatment for adults with ADHD. This proof-of-principle study exploring a rt-fMRI neurofeedback training for the first time in adult ADHD demonstrates both that the methodology is generally feasible and that such a training targeting the dACC can significantly improve cognitive functioning. Further, the results suggest that self-regulation success can be predicted by working memory/inhibition capacities, therefore calling for more elaborate multifaceted interventional approaches. Due to the limited sample size in the current study, the clinical benefits of the novel approach need to be evaluated in future studies.

## Supporting Information

S1 Fig Neurofeedback display. Participants in the neurofeedback group were instructed to performed mental calculations at varying levels of difficulty to achieve up-regulation of their activation level within dACC target regions. They were cued to either rest (A), reach a medium (B), or high difficulty level (C) by adapting their mental-calculation task performance. Neurofeedback participants were able to monitor their dACC activation levels on the thermometer (activation level represented by filled grey squares, (D) [shown here only for the high activation-level condition]), while control participants saw the same thermometer display without feedback information.
(TIF)

S2 Fig Indices of individual regulation success. To evaluate individual performance three different performance indices were computed: an index of *general task performance* (mean activation level across sessions (A)), an index of *improvement over sessions* (increase in activation level over sessions (B)), and an index of *improvement in differential modulation* (increase in increase in differential activation between task conditions (C)).
(TIF)

S3 Fig Motion and data quality during MRI training. Neurofeedback participants (blue bars) showed significantly reduced motion (A), marked with an asterisk) and significantly increased fMRI data quality as measured by tSNR (B), marked with an asterisk) in comparison to control participants (green bars). In both groups worse motion control was linked to considerably reduced tSNR (each dot represents an individual functional run (C)).
(TIF)

S4 Fig Questionnaire of Current motivation. Across all participants, perceived challenge decreased significantly over time (A), with level of interest decreasing significantly as well (B), and mastery confidence increasing over time (C). Incompetence fear did not change over time (D). There were no group differences that developed over time. The only difference between groups was that neurofeedback participants perceived the training generally as posing a higher challenge when compared to the control group ((A) marked with an asterisk).
(TIF)

S1 File CONSORT checklist. This list summarizes the information provided regarding how this exploratory randomized, single-blinded study was designed, analyzed and interpreted.
(PDF)

S2 File Ethics proposal. The ethics proposal approved by the local Medical Ethics Committee.
(PDF)

S3 File Training data. This file contains the activation levels (beta estimates) per individual and functional run during training, derived from the individually defined dACC target regions.
(CSV)

S4 File Behavioral assessment data. This file contains all clinical and neuropsychological data (per individual) from the behavioral assessment administered pre- and post-training, as well as the motivational data collected during each training session.
(CSV)

S1 Table dACC target regions. The x, y and z coordinates in Talairach space of the individual dACC target regions are shown per subject and session. SD = standard deviation.
(PDF)

S2 Table Predictors of dACC self-regulation (within group). Significant effects (p ≤ 0.05) are printed bold and marked with an asterisk. WAIS = Wechsler Adult Intelligence Scale, ADHD = Attention Deficit Hyperactivity Disorder, VC = Vocabulary, BD = Block Design, MSIT = Multi Source Interference task, SA-DOTS = Sustained Attention DOTS task, SART = Sustained Attention to Response Task, WM = Working memory, DS = Digit Span, LNS = Letter Number Sequencing.
(PDF)
