# Supplementary figures and images for: fMRI Neurofeedback Training for Increasing Anterior Cingulate Cortex Activation in Adult Attention Deficit Hyperactivity Disorder. An Exploratory Randomized, Single-Blinded Study
Source: PLoS One. 2017 Jan 26;12(1):e0170795. doi: 10.1371/journal.pone.0170795 (PMC5270326; doi:10.1371/journal.pone.0170795)

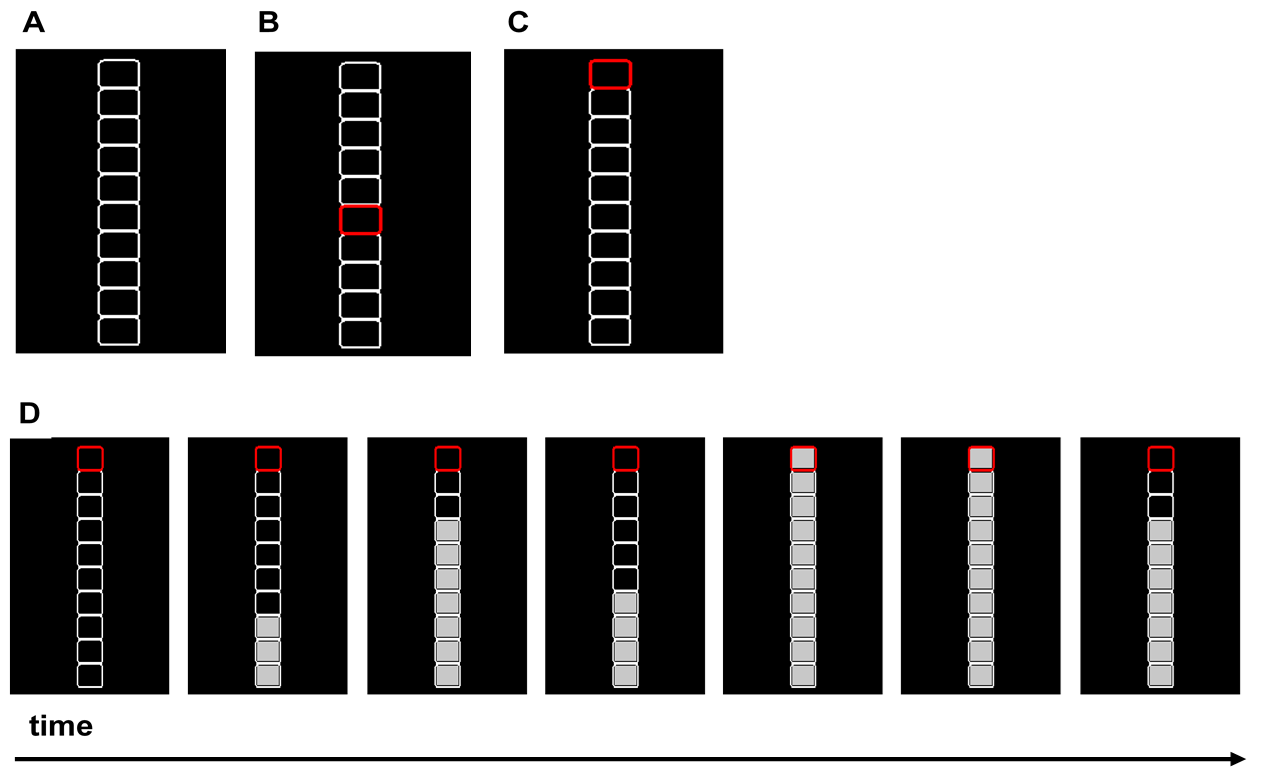

Supplement: S1 Fig — Participants in the neurofeedback group were instructed to performed mental calculations at varying levels of difficulty to achieve up-regulation of their activation level within dACC target regions. They were cued to either rest (A), reach a medium (B), or high difficulty level (C) by adapting their mental-calculation task performance. Neurofeedback participants were able to monitor their dACC activation levels on the thermometer (activation level represented by filled grey squares, (D) [shown here only for the high activation-level condition]), while control participants saw the same thermometer display without feedback information. (TIF) [file pone.0170795.s001.tif]

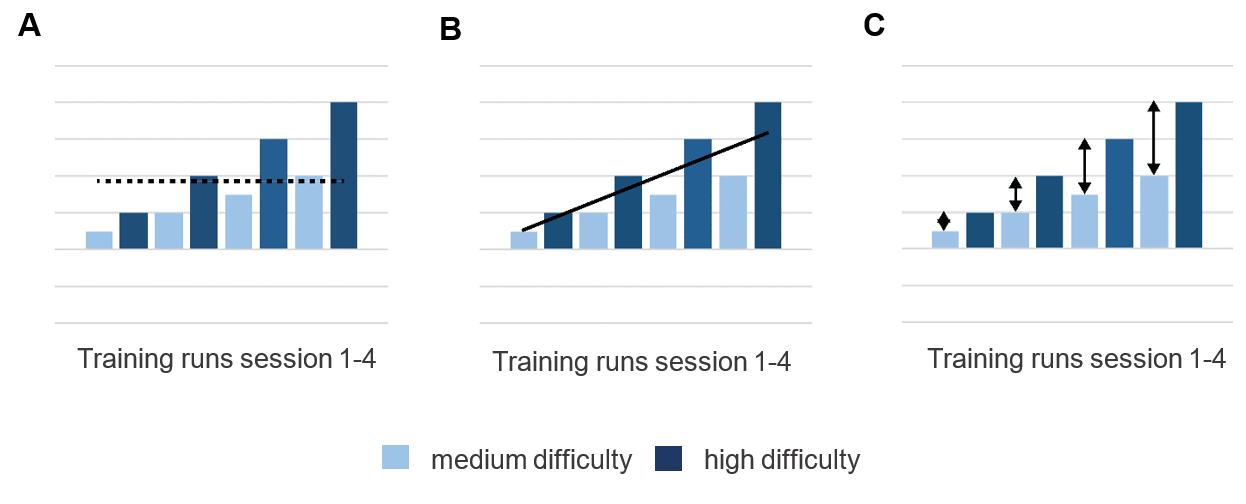

Supplement: S2 Fig — To evaluate individual performance three different performance indices were computed: an index of general task performance (mean activation level across sessions (A)), an index of improvement over sessions (increase in activation level over sessions (B)), and an index of improvement in differential modulation (increase in increase in differential activation between task conditions (C)). (TIF) [file pone.0170795.s002.tif]

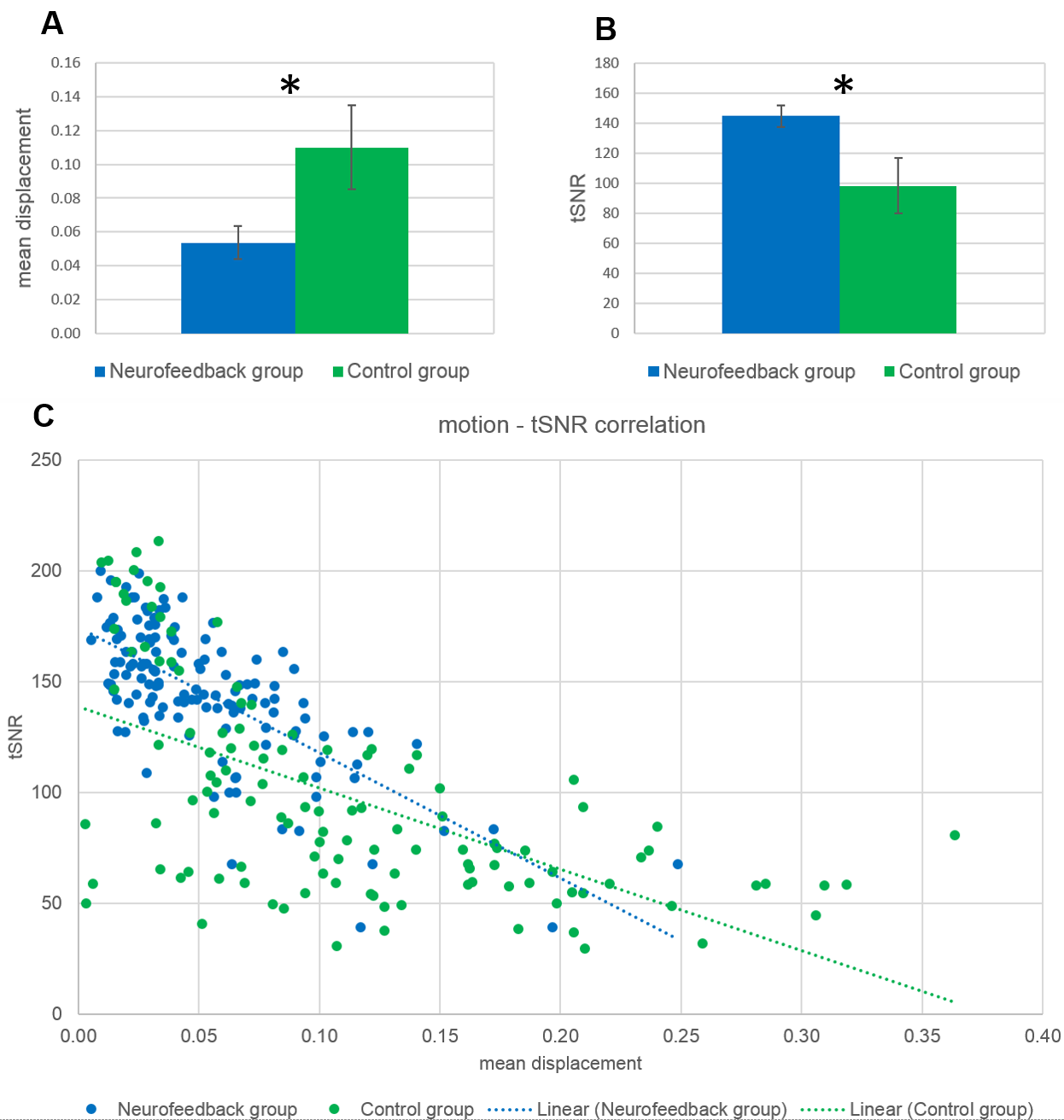

Supplement: S3 Fig — Neurofeedback participants (blue bars) showed significantly reduced motion (A), marked with an asterisk) and significantly increased fMRI data quality as measured by tSNR (B), marked with an asterisk) in comparison to control participants (green bars). In both groups worse motion control was linked to considerably reduced tSNR (each dot represents an individual functional run (C)). (TIF) [file pone.0170795.s003.tif]

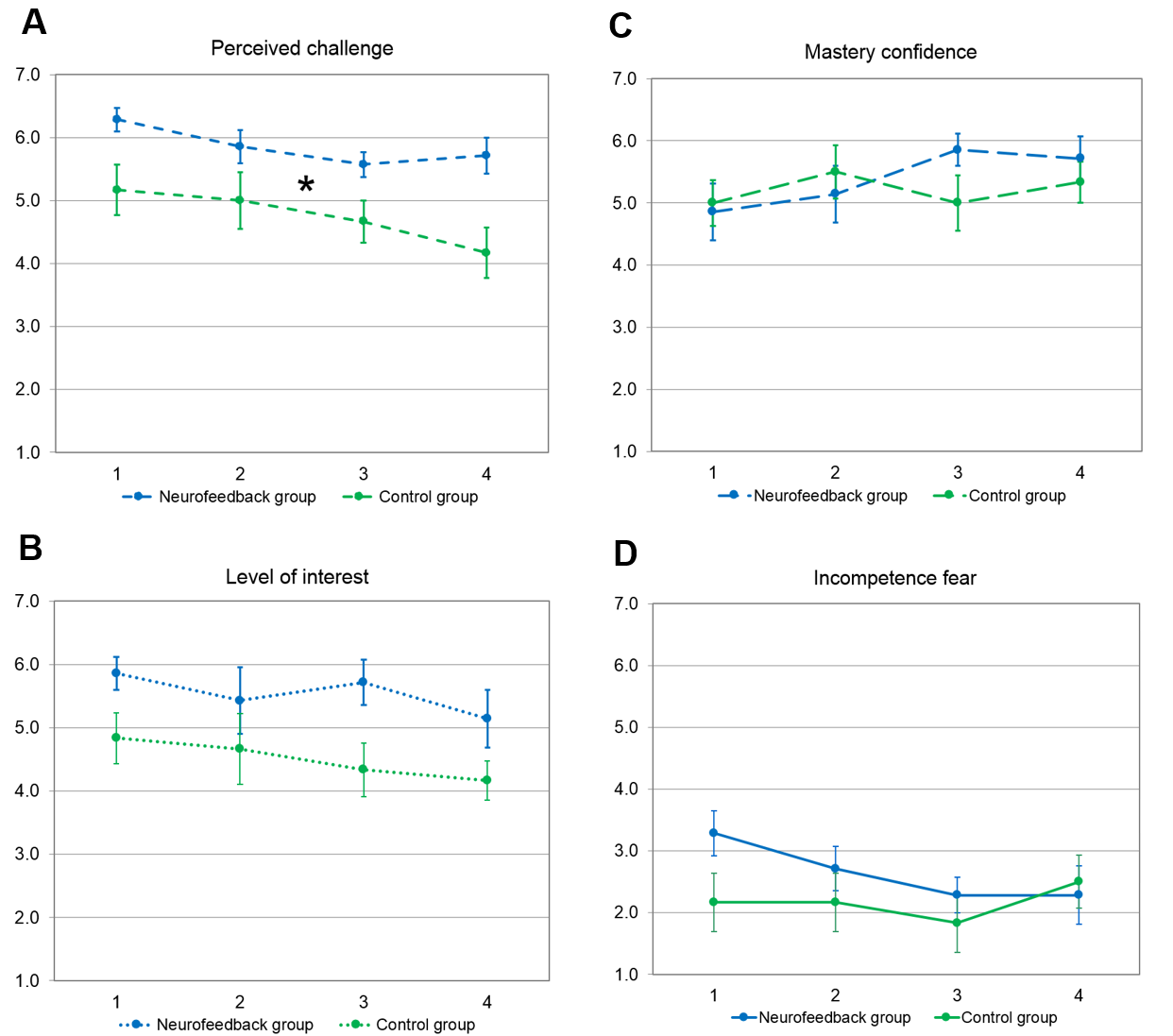

Supplement: S4 Fig — Across all participants, perceived challenge decreased significantly over time (A), with level of interest decreasing significantly as well (B), and mastery confidence increasing over time (C). Incompetence fear did not change over time (D). There were no group differences that developed over time. The only difference between groups was that neurofeedback participants perceived the training generally as posing a higher challenge when compared to the control group ((A) marked with an asterisk). (TIF) [file pone.0170795.s004.tif]
