# Supplementary material for: fMRI Neurofeedback Training for Increasing Anterior Cingulate Cortex Activation in Adult Attention Deficit Hyperactivity Disorder. An Exploratory Randomized, Single-Blinded Study
Source: PLoS One. 2017 Jan 26;12(1):e0170795. doi: 10.1371/journal.pone.0170795 (PMC5270326; doi:10.1371/journal.pone.0170795)
Supplement: S2 File — The ethics proposal approved by the local Medical Ethics Committee. (PDF) [file pone.0170795.s006.pdf]

ADHD and fMRI-neurofeedback:

A randomized controlled exploratory study on the feasibility of self-modulating anterior cingulate cortex activation levels

ADHD and fMRI-neurofeedback: A randomized controlled exploratory study on the feasibility of self-modulating anterior cingulate cortex activation levels

**Short title**	ADHD and fMRI-neurofeedback
**Version**	3
**Date**	September 24th, 2012
**Principal Investigator**	Prof. dr. Jan K. Buitelaar
**Research team**	Prof. dr. Jan K. Buitelaar, UMC St. Radboud/ Karakter<br>Prof. dr. Rainer Goebel, FPN-CN Maastricht University<br>Drs. Anna Zilverstand, FPN-CN Maastricht University<br>Dr. Bettina Sorger, FPN-CN Maastricht University<br>Dr. Dorine Slaats, Karakter<br>Dr. Cees Kan, UMC St Radboud<br>Prof. dr. David Norris, FC Donders Centre for Cognitive Neuroimaging
**Sponsor**	External Funding SmartMix Program NWO
**Independent Physician**	Dr. Joost Janzing, UMC St Radboud
**Laboratory sites**	Department of Cognitive Neuroscience, UMC St Radboud and FC Donders Centre for Cognitive Neuroimaging (data-collection)<br>Department of Psychiatry, UMC St Radboud and Karakter (recruitment)

Name	Signature	Date
**Head of Department Cognitive Neuroscience:**<br>Prof. dr. Guillen Fernandez		
**Principal Investigator:**<br>Prof. dr. Jan k. Buitelaar		

Table of contents

1. Background 6
ADHD 6
Current treatment of ADHD 6
fMRI-neurofeedback 7
fMRI research on ADHD 8
2. Objectives 9
3. Study Design 10
4. Study Population 11
Recruitment 11
Inclusion Criteria 11
Exclusion Criteria 12
Randomization procedure 12
5. Treatment of Subjects 13
6. Methods 16
Location 16
Overview Performed Measurements 16
Summary Measurements 21
Withdrawal of individual subjects 22
Premature termination of the study 22
7. Safety Reporting 23
Section 10 WMO event 23
Adverse events 23
Serious adverse events 24
8. Statistical Analysis 25
9. Ethical Considerations 26
Regulation statement 26
Benefits and risks assessment 26
Compensation for injury 26
Incentives 26
10. Administrative Aspects and Publication 27
Handling and storage of data and documents 27
Amendments 27
Annual progress report 27
End of study report 28
Public disclosure and publication policy 28
11. References 29

Summary

Title: ADHD and fMRI-neurofeedback: A randomized controlled trial to explore the effects of self-modulating anterior cingulate cortex activation levels

Background: First explorative studies have shown that fMRI-neurofeedback training has beneficial effects on the symptoms of patients with disorders as diverse as chronic pain, tinnitus, schizophrenia, psychopathy, Parkinson and stroke. No such study has been performed so far with an ADHD patient group. As a dysfunction of the anterior cingulate cortex (ACC) is hypothesized to underlie the symptoms of ADHD, this study is designed to examine the efficacy and safety of an fMRI-neurofeedback training in self-modulating the ACC activation level in adults with ADHD for the first time, as well as to study the role of the ACC in the pathophysiology of ADHD.

Objectives:

(1) Primary objective: investigating whether the modulation of the ACC activation level through fMRI-neurofeedback training reduces ADHD symptoms and improves cognitive functioning.
(2) Secondary objective: investigating if abnormal activation levels of the ACC in ADHD patients play a causal role in ADHD pathophysiology.

Study design: Randomized controlled treatment study with blinding of participants.
Study population: 20 subjects with ADHD (age > 18, IQ > 100).
Intervention: 10 subjects with ADHD receive 6 sessions with fMRI-neurofeedback from the dorsal anterior cingulate cortex, and 10 subjects with ADHD receive 6 sessions with fMRI-neurofeedback from a control region.
Main study parameter: ADHD-DSM-IV rating scale (rated blindly).

Nature and extent of the burden and risks associated with participation, benefit and group relatedness: Risks or side-effects are not expected. The burden for the ADHD subjects consists of an intake, pre- and post-treatment assessments (3 visits of ~75 minutes), and additionally 6 visits (~75 minutes) for the fMRI-neurofeedback training. The intake and pre-assessment carry the same burden as the treatment as usual. The benefit involves the possible beneficial effect of the fMRI-neurofeedback training on ADHD symptoms.

1. Background

ADHD

Attention-deficit/hyperactivity disorder (ADHD) is the most commonly diagnosed childhood–onset neuropsychiatric disorder. It is characterized by inattention, hyperactivity, and impulsivity, either alone or in combination (American Psychiatric Association, 2000). While 5 to 10% of all school-aged children in European countries are affected, the disorder may persist into adulthood in one third of the cases or more (Spencer, Biederman, & Mick, 2007). Individual and societal costs include impaired academic, occupational, and social functioning, increased rates of substance abuse, traffic accidents, and persistent neuropsychological impairments (Biederman, 2004; Secnik, Swensen, & Lage, 2005). Because of the severity and enduring nature of the functional impairments associated with ADHD, a substantial amount of scientific effort has been directed on *understanding the pathophysiology* of ADHD and identifying *effective treatments* of ADHD. Both topics will be addressed by this study.

Current treatment of ADHD

While first-line treatment for children with ADHD is the prescription of psychostimulants (i.e., methylphenidate or dextro-amphetamine), there is no approved first-line treatment for treating adults with ADHD in the Netherlands. In general drug treatment in adults has proven to be less effective than drug treatment in children. A review on the efficacy of medications in ADHD adults concluded that the response rates to medication were only 50 % when stimulants were prescribed and as low as 20 % when nonstimulants were taken (Faraone & Glatt, 2010). Common adverse effects of stimulants include vertigo, decreased appetite, weight loss, mood lability, tension, and depression (Santosh, Sattar, & Canagaratnam, 2011). Adverse effects are especially problematic because treatment is generally long term, as symptoms of ADHD reappear after discontinuing drug treatment. Also, while medication does improve attention, it is still unclear if it has a positive effect on academic, occupational and social functioning in adults with ADHD (Santosh, Sattar, & Canagaratnam, 2011). Data on the efficacy of alternative treatments as for example cognitive behavioral treatment in ADHD in adults is still preliminary (Antshel et al., 2011).

One proposed alternative treatment without adverse side effects is EEG-neurofeedback. Neurofeedback in general is defined as a procedure during which a participant learns self-control over some aspect of neuronal functioning of his brain through getting feedback on it. The aim in general is to normalize a deviant neuronal pattern, which should also lead to a reduction of the symptoms of the patient. The goal in ADHD patients is to teach participants how to control certain EEG signals that are an indicator of alertness (Sterman, 1996). Recent reviews on EEG-neurofeedback have concluded that preliminary results are very promising regarding the reduction of ADHD symptoms and improvement of cognitive deficits (Fox, Tharp, & Fox, 2005; Heinrich, Gevensleben, & Strehl, 2007; Hirshberg, 2007; Loo & Barkley, 2005; Rossiter, 2004).

These results have spurred interest into the development of other neurofeedback methods as well, as for example neurofeedback based on functional magnetic resonance imaging (fMRI). The advantage of fMRI-neurofeedback over EEG-neurofeedback may be the higher spatial resolution and full brain coverage achieved with fMRI, and therefore also a possible faster treatment response. Functional magnetic resonance imaging (fMRI) was the first non-invasive imaging method to provide us with high spatial resolution measurements of blood oxygenation as an indirect measure of neuronal activity (Bandettini, Birn, & Donahue, 2000), and has thus advanced our understanding of the human brain considerably over the last 20 years.

fMRI-neurofeedback

fMRI is a method with a high degree of patient safety, there is no evidence for hazards associated with increasing exposure (Hawkinson et al., 2011; Schenck, 2000). Since the mid-1990s several research groups have been working on the development of fMRI real-time techniques, techniques which allow for immediate data processing and data analysis during fMRI scanning. Current real-time fMRI procedures include most state-of-the-art data preprocessing and analysis steps of its classical offline counterpart (Weiskopf, in press; Weiskopf et al., 2007). Importantly, it has been show that real-time fMRI setups have a safety level similar to a normal fMRI setup (Hawkinson et al., 2011).

Many studies have focused on the general feasibility of fMRI-neurofeedback. As in EEG-neurofeedback the goal is to learn how to voluntarily modulate some aspect of neuronal activity. Numerous studies have shown that participants are indeed able to control their brain activation patterns in very specific ways, and that participants profit

from using fMRI-neurofeedback when learning how to do this (Weiskopf, in press). Importantly, specific behavioral effects are correlated with specific changes in brain activation patterns (Weiskopf, in press). Also, first studies with patients indicate that patients with disorders as diverse as chronic pain, tinnitus, schizophrenia, psychopathy, parkinson and stroke may experience some relief from their symptoms after a fMRI-neurofeedback training (deCharms et al., 2005; Haller, Birbaumer, & Veit, 2010; Ruiz et al., 2011; Sitaram et al., 2011; Subramanian et al., 2011; Veit, 2009).

fMRI research on ADHD

As the general goal in patient studies is the normalization of the brain activation patterns which are linked to the behavioral symptoms of this disorder, one of the most important considerations for setting up a fMRI-neurofeedback training is which aspect of the brain activation patterns are most closely linked to the behavioral symptoms. By convergent data from a variety of sources, including neuroimaging, neuropsychological, neurochemical and genetic studies, the core symptoms of ADHD have been linked to abnormalities in the functioning of frontal, cingulated and parietal cortical brain regions (Bush, 2011). The brain region that has been most consistently linked to ADHD pathology across all these studies is the dorsal anterior cingulate cortex (Bush, 2011). Long term structural changes have been shown in this region (Amico, Stauber, Koutsouleris, & Frodl, 2011; Konrad et al., 2010; Makris et al., 2007; Seidman et al., 2011; Seidman et al., 2006), and fMRI research has consistently found a characteristic pattern of hypoactivation when subjects are performing tasks which are typically challenging to them, e.g. interference task, continuous performance test, switch task, response inhibition task (Bush, 2011; Bush et al., 1999; Bush et al., 2008; Cubillo et al., 2010; Dickstein, Bannon, Castellanos, & Milham, 2006; Schneider et al., 2010). It has also been shown that this hypoactivation normalizes after successful treatment with ADHD medication (Bush et al., 2008). Normalization of this pattern of hypoactivation thus seems to be a crucial aspect in treatment success.

In the proposed study we want to train ADHD patients how to voluntarily upregulate the activation level of the dorsal anterior cingulated cortex. Several previous studies with healthy participants as well as with pain patients has already shown that in general it is possible to upregulate the activation level of the anterior cingulate cortex (deCharms et al., 2005; Hamilton, Glover, Hsu, Johnson, & Gotlib, 2011; Weiskopf et al., 2003).

2. Objectives

The primary objective of this study will be to critically evaluate if the fMRI-neurofeedback training is successful in reducing ADHD symptoms and improving cognitive functioning. Thus, the first goal is to show that ADHD patients are able to voluntarily modulate their individual anterior cingulate cortex activation level. Secondly, it has to be demonstrated that this modulation has a specific influence on ADHD symptoms and the performance during cognitive tasks. Finally, it has to be critically evaluated which outcome measures are positively modulated, and which are the moderating factors for treatment success. All the cognitive tasks that have been included in the design are also possible moderating factors.

The secondary objective of this study is further enhancing the understanding of the pathophysiology of ADHD. Neurofeedback studies in general are seen as an excellent tool for investigating the causal influence of abnormal brain activation levels. As the regional brain activation level is manipulated in a neurofeedback experiment, the ADHD symptoms and cognitive functioning become the outcome measure. If one would succeed in influencing behavior through manipulating brain activation patterns, this would be strong evidence for a causal role of the abnormal activation levels of the anterior cingulate cortex in the pathophysiology of ADHD.

<u>Summary objectives</u>

(1) Primary objective: investigating whether the modulation of the ACC activation level through fMRI-neurofeedback training reduces ADHD symptoms and improves cognitive functioning.

(2) Secondary objective: investigating if abnormal activation levels of the ACC in ADHD patients play a causal role in ADHD pathophysiology.

3. Study Design

This study is a randomized controlled trial (RTC) with blinding of the participants and blinding of all raters. ADHD adults will be blindly allocated to one of the following two groups by a restricted randomization technique (see recruitment section):

1. fMRI-neurofeedback training, feedback from <u>dorsal anterior cingulate cortex (n=10)</u>
2. fMRI-neurofeedback training, feedback from <u>control brain region (n=10)</u>

Each group will receive the same screening, pre- and post-assessment and 6 fMRI-neurofeedback training sessions. The duration of each fMRI-neurofeedback training session will be approximately 75 minutes. The frequency will be weekly sessions. The duration of the experiment for each participant from the selection until the last session will be approximately 9 weeks.
The control group will receive exactly the same as the fMRI-neurofeedback group, except for the feedback. The control group will receive feedback from a control brain region, which will be selected such that it is activated by different tasks than the dorsal anterior cingulate cortex. This selection will be made based on literature research, previous data, and data from an ongoing pilot experiment.

Timeline study design

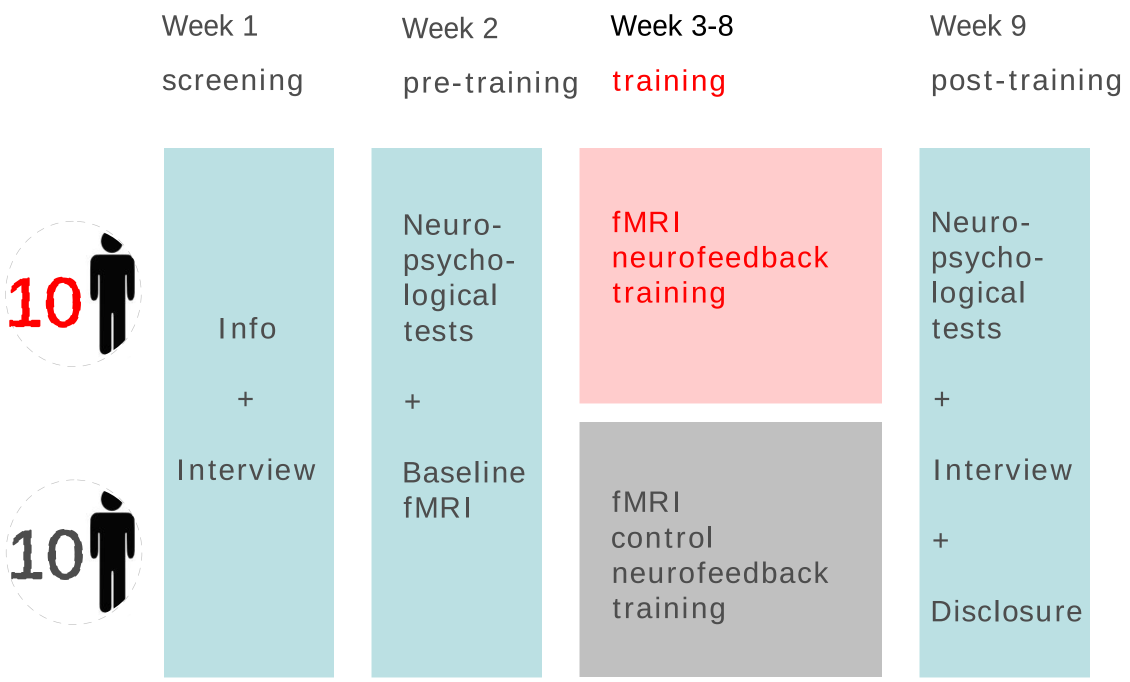

4. Study Population

Recruitment

Participating ADHD patients will be recruited among patients already known to Karakter and among new referrals and patients already known to the ADHD program of the Department of Psychiatry, UMC St Radboud. We will also recruit patients from the patients associations Balans and Impuls through advertisements in their magazines, and by advertisements on internet on various websites. Patients who are interested and think they meet the criteria on the flyer "E3.Oproep ADHD fMRI neurofeedback studie" (see appendix to this protocol) are invited to send an email to ADHD-MRINF@donders.nl . They will be contacted by phone for further information about the study. If they stay interested, they will be sent detailed written information about the study "E1.Informatiebrief ADHD fMRI neurofeedback", "E1.Informatiebrochure fMRI onderzoek", "E1.Verzekeringsinformatie", and attached informed consents "E2.Toestemmingsverklaring ADHD fMRI Neurofeedback studie", "Written Consent fMRI Nijmegen", (see appendix to this protocol). They can think about participation for 2 weeks.

When the informed consents are signed, and received by the research team, the investigator will sign the informed consents and will return a photocopy to the participant. If the investigator does not receive the informed consent, the patient will be contacted to ask if they want to participate and again asked to send back the informed consent. Subsequently, the general practitioner and the possible involved therapist/psychiatrist (for the ADHD) will be informed about the participation on the study. If there is objection to inform them, the patient cannot participate in the study. See for the written information to general practitioner and therapist, "K5.Uitleg participatie 'Project ADHD & FMRI Neurofeedback' Behandelaar" (see appendix to this protocol".

Inclusion Criteria

- Diagnosis of ADHD according to the DSM-IV TR criteria (American Psychiatric Association, 2000)
- Age > 18
- Psychopharmaca-naïve or –free, or being on a fixed dose of medication for the study period (patients may pro-actively opt for non-medical treatment)
- Passing fMRI screening criteria, which consist of the following:

  - no previous brain operation
  - no epilepsy
  - no implants as for example pacemakers or an implanted insulin pump
  - no metal parts in the body (protheses, implants, clips on blood vessels, spiral, or other metal objects except teeth fillings and connectors)
  - no claustrophobia
  - no pregnancy
- IQ > 100 according to block design and vocabulary test WAIS-III-NL (Uterwijk, 2005)

Exclusion Criteria

- Participation in another clinical trial simultaneously
- Previous participation in neurofeedback training
- Other significant medical condition (e.g. neurological, heart or vascular diseases) or regular use of medication other than psychostimulants if the dosis of medication is not fixed for the study period
- Current diagnosis of one or more Axis I diagnosis other than ADHD according to the DSM-IV TR criteria (American Psychiatric Association, 2000) (e.g. depression, psychosis, tics, autism, eating disorders or behavioural disorders)
- Current alcohol or drug abuse according to the DSM-IV TR criteria (American Psychiatric Association, 2000)

Randomization procedure

Balancing potential confounding factors is of special importance in a study with a sample size as small as the proposed study. To achieve this we will apply a restricted randomization technique called sequential balancing, a form of minimization. This has been shown to be efficient in balancing several factors in studies with a small sample size, as balance is achieved for each factor separately, instead of for the combination of the factors (Borm, Hoogendoorn, den Heijer, & Zielhuis, 2005; Scott, McPherson, Ramsay, & Campbell, 2002). The following two minimization factors will be used:

- Dose of psychostimulant medication (levels: no dose, low dose, high dose)
- ADHDDSM-IV rating scale score Dutch version of this scale (Kooij et al., 2004) (levels: high or low ADHD score)

5. Treatment of Subjects

The fMRI-neurofeedback training will proceed as follows. Before the training the subjects will be suggested a set of cognitive strategies that they may use during the neurofeedback training (the same set of strategies in both groups). These cognitive strategies will be derived from research on which mental tasks activate the neurofeedback and the control region. Towards the patients it will be stressed that they are also always free to choose any other strategy that seems to work, and that they should be guided by the feedback in the selection of their strategy.

At the beginning of each scanner session patients will given ear plugs to protect them against the scanner noise. Additionally, they will be given customized headphones which enable them to communicate with the researchers at any point of time of the scanning session. Also, they will receive an alarm button, which they can press at any point of time if they wish to interrupt the scanning session immediately. The participants will be moved into the fMRI scanner lying on their back with their head fixated by small foam cushions to prevent excessive head movement. In the scanner they will always have the projection of a full computer display visible in a mirror, which is mounted directly in front of their eyes. This display will later be used to present them with the feedback regarding their own brain activation (see figure below for setup).

The training scan session will then start with the 10 minute multisource interference task during which the participants will have to indentify a target number 1, 2, or 3 in a adapted Flanker task by button presses. The brain activation measured during this interference task will be used to functionally localize the feedback target region immediately after this functional scan. The localization of the feedback target region will be done by researchers with ample experience in localizing feedback target regions in general, as well as with localizing the ACC based on the interference task proposed in this study. The necessary analysis will be performed during the following 8 minute anatomical scan. The subject will not have to spend any additional time in the scanner, as the anatomical scan is needed for co-registration of the data in any case. During the anatomical scan the patient will be told to relax, and watch a short silent video that will be presented on the display to divert them from the scanner environment.

During the neurofeeback training the patients will see their own current brain activation level (BOLD % signal change) from the individually defined feedback target region in the dorsal anterior cingulate cortex (experimental group), or the control region (control group). They will see the current activation level presented with a simple a visual

thermometer display, which will be continuously updated (every 1.5 second), as shown in the figure below. The thermometer will be individually scaled according to the activation level measured during the previous localizer task. Participants will be instructed to increase and maintain activation levels to a 50% or to a 100% level, depending on the target level indicated by the red box (see figure). The subjects will have no other task than to using mental strategies to manipulate their brain activation level and observe and reflect on the changes in the displayed activation level while they are doing this. A similar instruction has been successfully used in previous studies with healthy participants (Sorger, 2010).

The training will last approximately 30 minutes and consist of 3x 8 “neurofeedback” blocks of 30 seconds during which they will manipulate their brain activation, each neurofeedback block being followed by a resting period of 20 seconds. After a series of 8 blocks there will be a self-paced break. Finally, 8 “transfer” blocks will follow, during which subjects will be asked to apply whatever strategy they have learned previously, but now without receiving neurofeedback. They will thus be asked to transfer what they have learned during the training to a situation without neurofeedback.

In our opinion the setup and procedure during the fMRI neurofeedback training is quite comparable to the setup and procedure used in cognitive neuroscience experiments. The main difference to these experiments is that during the neurofeedback training the participants are less restricted regarding the mental tasks that they perform. To take into account the possible shorter attention span of the target groups we reduced the length of the session in comparison to previously performed sessions with healthy subjects. Regarding strain from participating in multiple fMRI sessions there is one previous fMRI neurofeedback training study that systematically investigated the safety of patients by analyzing the incidence and severity of adverse events. This study implemented a very similar training as the one proposed here, asking the patients to participate in six to nine repeated fMRI neurofeedback sessions of one hour length. The most frequently reported adverse events in this patient group were a) somnolence and b) increased pain (Hawkinson et al., 2011). As the study was conducted with a group of refractory pain patients, both types of adverse events were not reported more frequently in the neurofeedback group than in the baseline group. There was no overall increased number of adverse advents in the neurofeedback group compared to the baseline group (Hawkinson et al., 2011).

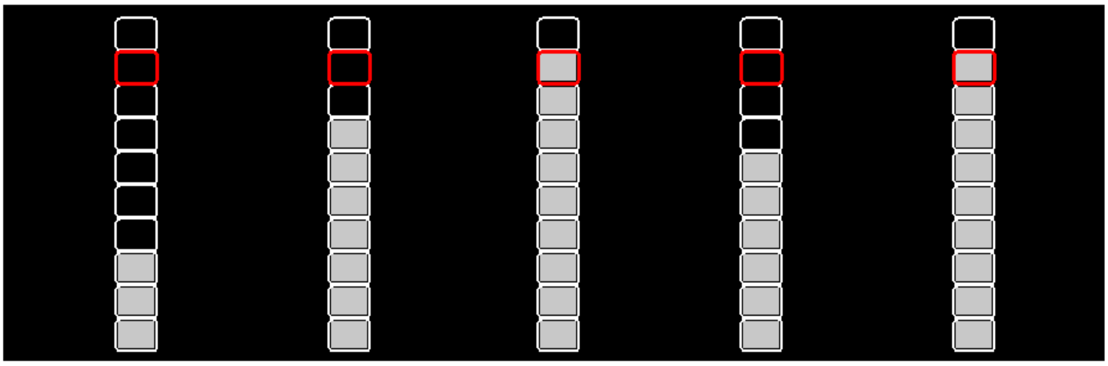

Figure: Thermometer display for the presentation of the neurofeedback

The scale of the thermometer will be adjusted based on the activation level during the localization task such that the maximum of the scale is a realistic target. The participant will be asked to aim a reaching the target level indicated by the red box, which might be either 50 % or 100% of the maximum depending on the condition. Five different frames with varying activation levels are shown in the figure.

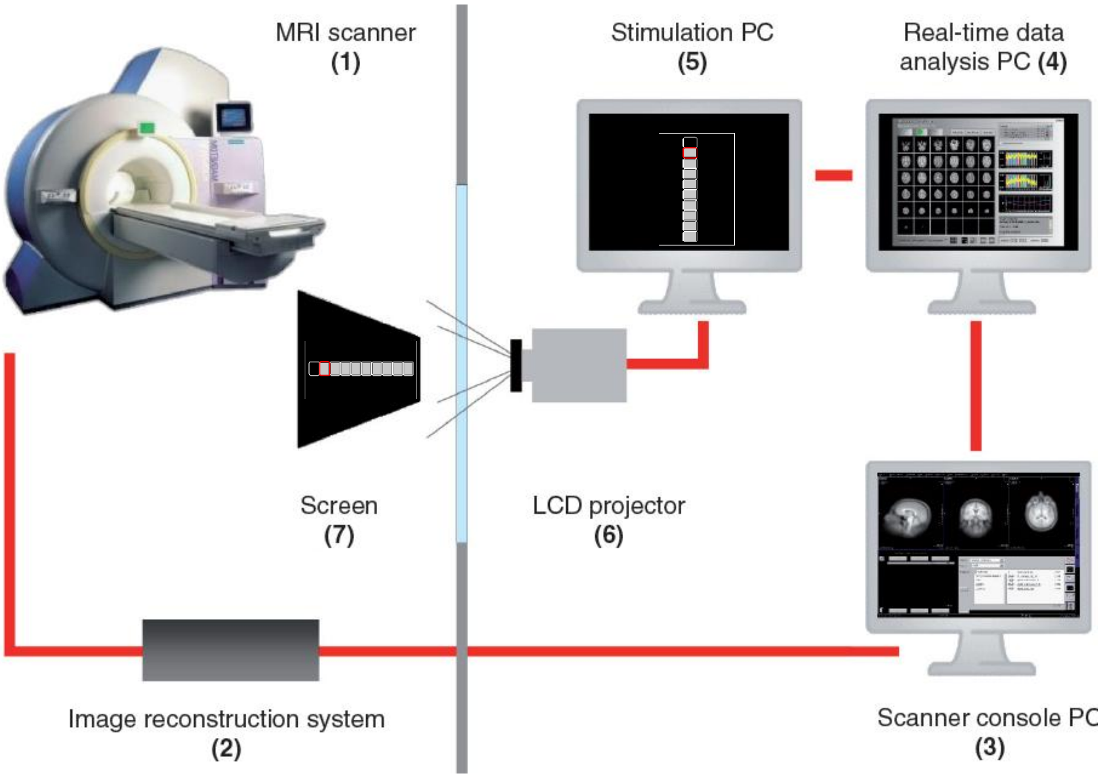

Figure: Technical setup during the fMRI neurofeedback training

The visual feedback presented on the stimulation PC will be projected onto a mirror mounted directly in front of the eyes of the participant.

6. Methods

In the following part, we will describe detailed the whole procedure from beginning till end, including the rationale for the proposed measurements.

Location

Selection of subjects, screening for eligibility and assessments, and the fMRI-neurofeedback training will be performed at the FCDC. All fMRI assessments will be performed on a Siemens T3 Magnetom MRI scanner.

Overview Performed Measurements

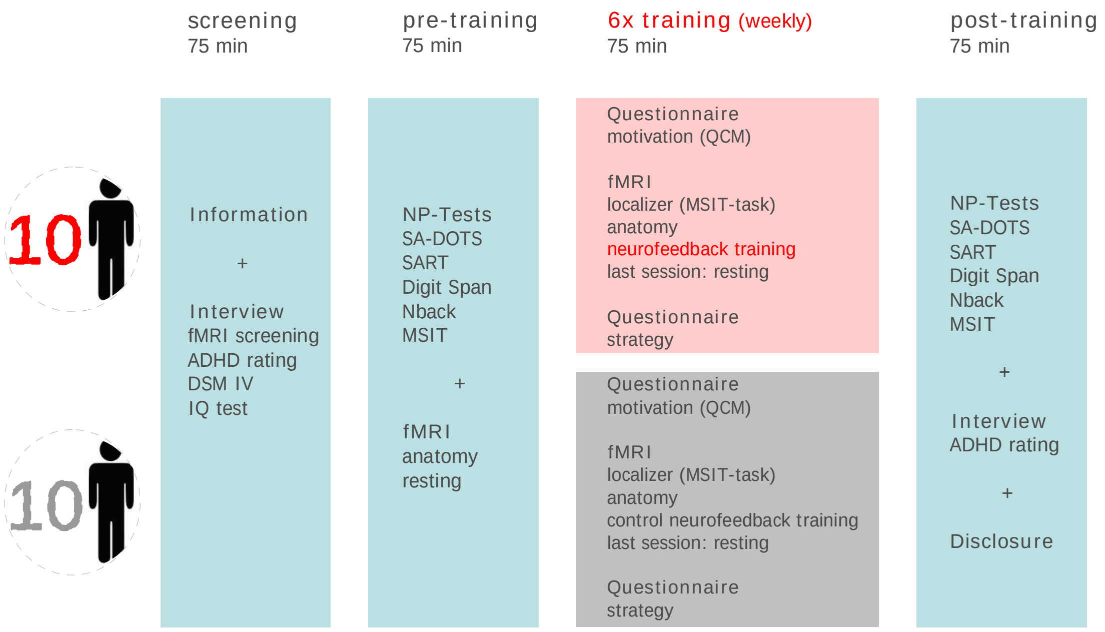

During the **screening session** (~ 75 minutes) the inclusion/ exclusion criteria will be assessed (DSM IV interview, fMRI screening interview, age, IQ Test, use of medication, significant medical conditions, participation in therapy, and participation in other clinical trials). Also the following two baseline measurements will be made:

- A first assessment of the severity of ADHD symptoms the ADHD DSM-IV rating scale, which is a widely used instrument for determine the severity of ADHD, fully based on the DSM-IV (American Psychiatric Association, 2000; Kooij et al., 2005).

- To estimate the intelligence, a shortened version of the WAIS-III-NL (Uterwijk, 2005) will be administered; the Vocabulary test (~10 minutes) and the Block Design test (~10 minutes). Validity coefficients for the Vocabulary and Block Design scores relative to the full form are .88 for Verbal IQ and .83 for Performance IQ (Antshel et al., 2007).

After the screening session randomization will be performed. Randomization will be used to avoid bias in the assignment of subjects to treatment, to increase the likelihood that known and unknown factors (expectations of the therapy, motivation etc.) are evenly balanced across treatment groups and to enhance the validity of statistical comparisons across treatment groups. Randomization will be stratified according to ADHD symptom score and IQ score.

An elaborate assessment of the ADHD symptoms will take place during the **pre-training session** (~75 minutes). During this session a neuropsychological assessment and a resting-state fMRI measurement will be completed. The following neuropsychological tests will be used:

Meta-analytic studies of neuropsychological function in ADHD report moderate to large effect sizes a sustained attention deficit in ADHD (Willcutt, Doyle, Nigg, Faraone, & Pennington, 2005). To assess sustained attention we will use the Sustained Attention dots task (SA-DOTS) as well as the Sustained Attention to Response Task (SART). Both tasks have been shown to discriminate ADHD patients from healthy subjects (Marchetta et al., 2008; Slaats-Willemse et al., 2005; Smilek et al., 2010).

- The SA-DOTS (~15 minutes) is the so-called continuous performance task from the ANT, a computerized neuropsychological test battery (De Sonneville, 1999). During the SA-DOTS task the subject is presented random spatial dot patterns with 3-5 dots. The subject needs to press yes for a 4 dot pattern and no for a 3, or 5 dot pattern. Premature responses, false alarms, misses, the reaction time and the standard deviation of the reaction time are indicators of

the ability to maintain attention over time. The test-retest reliability of the SA-DOTS is excellent (0.93 – 0.97, personal correspondence with Leo de Sonneville), which makes the test suited for measuring differences in the ability to sustain attention before and after the fMRI-neurofeedback training.

- During the SART (~10 minutes) single digits are presented at a rate of just over one per second. Participants are told to press a button to every number, except if that number is 3. As the task is repetitive and apparently easy, it requires participants to maintain attention.

Another neuropsychological function that has been implicated in the ADHD is working memory. Differences to healthy participants have been found for verbal as well a spatial working memory, meta-analytic findings show an effect size of 0.41 to 0.51 for ADHD versus non-ADHD (Willcutt, Doyle, Nigg, Faraone, & Pennington, 2005). Additionally, working memory capacity has been linked to successful learning during neurofeedback trainings (Hammer et al., 2012). We will thus assess verbal working memory with the Digit Span subtest of the WAIS-III-NL and visuo-spatial working memory using a visuo-spatial Nback task.

- During the Digit Span (~10 minutes) test a sequence of digits is presented orally. The digit span is then measured by forward- and reverse-order (backward) recall of the digit sequences. Backward recall is interpreted as a measure of working memory. In the standard digit span test, the sequences are presented with increasing length and testing ceases as soon as the participant makes two consecutive errors. We will use a slightly different procedure, which was developed recently: all possible trials are presented and a mean score across all trials is calculated (Woods et al., 2011). Test-retest reliability for this procedure has been shown to be very good (0.83 for backward span)(Woods et al., 2011).
- To assess visuo-spatial working memory we use the NBack task (~10 minutes) (McElree, 2001). We will use a within-subjects design with three conditions: 2-, 3-, and 4-back, administrated in this fixed order.

Finally will assess cognitive interference, as subjects with ADHD usually underperform on tasks that require cognitive interference (Bush, 2011; Bush et al., 1999; Bush et al., 2008; Cubillo et al., 2010; Dickstein, Bannon, Castellanos, & Milham, 2006; Schneider et al., 2010). We will use the Multi-Source Interference Task (MSIT), a task that has

been especially developed such that it reliably and robustly causes very strong interference effects (Bush & Shin, 2006). It has also been shown that the MSIT discriminate ADHD patients from healthy subjects (Bush et al., 2008).

- During the MSIT (~10 minutes) subjects are presented with a visual display of a set of three numbers (0, 1, 2 or 3). They are asked to report, via button press, the identity of the number that differs from the other two numbers. Interference is caused between the value of the target number, the value of the accompanying numbers, the value of the location of the target number, and the location of the hand used to answer.

After the neuropsychological testing the pre-training session continues with a short fMRI scanning session (~20 minutes total), which includes an anatomical scan (~10 minutes) and a resting-state fMRI scan (~8 minutes). This resting-state fMRI scan will serve as a baseline measure to estimate the severity of the ADHD brain pathophysiology. Previous studies have shown that adults with ADHD show decreased coupling of the anterior cingulate cortex with other brain regions in comparison with healthy subjects (Castellanos et al., 2008). Additionally, this fMRI session will have the function of a practice session, during which subjects can get acquainted with the scanner environment, and the researchers who will be present during the later fMRI-neurofeedback training. If subjects are not comfortable in the scanner environment during the practise session they will be excluded from the study at this point.

- The fMRI session will proceed as follows. Subjects will be carefully instructed to remove all metal objects before entering the MRI scanner. To restrict head movements and to limit motion artefacts, the participant's head will be fixed by foam cushions and ear clamps positioned behind the neck and around the head. Participants will also be reminded to keep their head as still as possible. Headphones customized for MRI experiments will be inserted into the head coil and will provide isolation from scanner noise. These headphones will also be used to present instructions to the participants. To accustom subjects to the scanner noise the anatomical scan will be performed first.
- A high-resolution anatomical MRI scan will be acquired that is optimized for volumetric measurement of individual brain areas and of gray and white matter volumes and that can serve as anatomical reference for the functional scans. Subjects are told to relax and lie still during the anatomical scan.

- Second the resting-state scan will be performed. Resting-state fMRI measures fluctuations in the Blood-Oxygen-Level-Dependent (BOLD) signal in gray matter brain areas while the subject is at rest (not performing a task). Participants will be instructed to relax and remain still with eyes open for 8 minutes in the fMRI scanner.

During the second week the first **fMRI-neurofeedback training** session (~75 minutes) will take place. At the beginning of the session subjects are asked to fill in a short questionnaire on their current state of motivation regarding the training. Previous neurofeedback studies have shown that state of motivation is an important predictor of training success (Hammer et al., 2012; Nijboer, Birbaumer, & Kubler, 2010). Current state of motivation will be measured using an adapted version of Questionnaire for Current Motivation (QCM) (Nijboer, Birbaumer, & Kubler, 2010; Rheinberg, Vollmeyer, & Burns, 2001).

- The QCM (~10 minutes) consists of 18 short statements which have to be rated to which extend they apply on a 7-point Likert-type scale. Four factors of motivation (mastery confidence, incompetence fear, interest, and challenge) can be extracted from these 18 items.

After this the fMRI training session (~50 minutes total) will start. Each training session consists of three parts: a) localizer task (~10 minutes), b) anatomical scan (~8 minutes), and c) neurofeedback training (~30 minutes). Only during the sixth and last training session there will be an additional resting-state fMRI scan (~8 minutes) at the end of the session (see above).

In this study the designated feedback region will be defined individually at the beginning of each training. At the beginning of the session subjects will thus be asked to perform the Multi-Source Interference Task (MSIT) as the localizer task. The individual fMRI data collected during performance of localizer task will be analyzed immediately using fMRI online analysis software (TurboBrainVoyager 3.0, http://www.brainvoyager.com/products/turbobrainvoyager.html). The general procedure and safety precautions are the same as during the resting-state fMRI (see above). Visual stimuli will be presented on a screen that the participant will be able to see by means of a mirror attached to the head coil of the MR scanner.

The MSIT is chosen as the localizer task because subjects will already be acquainted with the task, as they have performed it during neuropsychological testing (see description above). It is also known that this task robustly and reliably activates the dorsal anterior cingulate cortex, also across sessions (Bush & Shin, 2006; Bush et al., 2008). Finally, this task has been successfully used in an ADHD patient group to localize dorsal anterior cingulate cortex (Bush et al., 2008).

After localizer task and the anatomical scan the fMRI-neurofeedback training will start (see description in section 5. treatment). The fMRI setup during the training will be the same as during the localizer task.

After each training subjects will be asked to fill in a short questionnaire (~10 minutes) on the strategies that they used during this training (Sorger, 2010). Participants will also be encouraged to practise and think about the mental strategies at home in-between the training sessions. Training sessions will take place once a week.

One week after the last training session the **post-training session** (~75 minutes) will take place. All the neuropsychological tests from the pre-training session will be repeated (SA-DOTS, SART, Digit Span, Nback-task, MSIT-task, see above), as well as the assessment of ADHD symptoms performed during the screening (see above).

Summary Measurements

Pre-Post training

- ADHD symptom score according to the DSM-IV
- Sustained attention score according to SA-DOTS and SART
- Working memory score according to Digit Span and Nback-task
- Anterior cingulate cortex activation connectivity during resting-state fMRI

fMRI-neurofeedback training sessions

- Current Motivation according to QCM
- Cognitive interference score according to MSIT
- Anterior cingulate cortex activation level during MSIT/ neurofeedback training
- Mental strategies used during neurofeedback task

Withdrawal of individual subjects

Subjects can leave the study at any time for any reason if they wish to do so without any consequences. The principal investigator can also withdraw a subject if:

- The investigator believes that for safety reasons (e.g. in the presence of adverse events) it is in the best interest of the subject to stop treatment.
- The subject is unwilling to cooperate for reasons not related to the trial treatment.

An intercurrent illness emerges of which the severity, duration, or required treatment violated the conditions of the trial.

Subjects withdrawn from the study for a medical reason will be followed until the adverse events have been resolved.

Premature termination of the study

The study will terminate prematurely if fMRI-neurofeedback does harm to the population of interest.

7. Safety Reporting

Section 10 WMO event

In accordance to section 10, subsection 1, of the WMO, the investigator will inform the subjects and the reviewing accredited METC if anything occurs, when the disadvantages of participation may be significantly greater than was foreseen in the research proposal. The study will be suspended pending further review by the accredited METC, except in so far, as suspension would jeopardize the subjects‟ health. The investigator will take care that all subjects are kept informed.

Adverse events

Adverse events are defined as any undesirable experience occurring to a subject during a clinical trial, whether or not considered related to the intervention. An adverse event can therefore be any unfavorable and unintended sign, symptom, or disease temporally associated with the intervention. All adverse events reported spontaneously by the subject or observed by the investigator or his staff will be recorded. Pre-existing complaints or symptoms that increased in severity or frequency during the treatment period will be recorded as well. For each adverse event the relationship to the intervention as judged by the investigator, will be recorded. If a subject discontinued the trial because of an adverse event this will be noted.

The FCDC has the following procedure for experiments involving healthy subjects:
1. If the researcher discovers accidently an anomalous region in the brain the only action he/she should take is to ask Prof. dr. Guillen Fernandez (neurologist) to look at the images. In this stage the subject is not informed about the finding.
2. Prof. dr. G. Fernandez will examine the images. He may contact a radiologist for a second opinion.
3. Prof. dr. G. Fernandez will inform the researcher about the medical relevance of the finding.
4. In case the finding represents a norm variant without any pathology the subject will not be informed.
5. In all other cases the subject will be informed by the principal investigator Prof. dr. Jan Buitelaar. He will contact the participant by phone and invite him/her for an

appointment. He will advice the subject to contact his/her family doctor for further medical examination. The participant will also be informed by a standard letter to the subject, signed by the principal investigator. A copy of the letter is also send to the family doctor of the subject.

Serious adverse events

The occurrence of an adverse experience that is fatal, life-threatening, disabling or requires in-patient hospitalization or causes congenital anomaly will be described as "serious" adverse event and would be notified in writing to the Commissie Mensgebonden Onderzoek Regio Arnhem Nijmegen.

All adverse events will be followed until they have abated, or until a stable situation has been reached. Depending on the event, follow up may require additional tests or medical procedures as indicated, and/or referral to the general physician or a medical specialist.

8. Statistical Analysis

Related to primary objective (1): Investigate whether fMRI-neurofeedback training reduces ADHD symptoms.

For the ADHD symptom score, the sustained attention measures, the working memory measures, and the fMRI resting state anterior cingulate cortex connectivity several independent 2 x 2- way ANCOVAs (one per outcome measure) will be conducted (two-tailed, significance level is set at 5%) with session (pre-vs. post-treatment) as within-subjects factor, group (fMRI-neurofeedback vs. control neurofeedback) as between-subjects factor, and age, sex and IQ as covariates.

For the cognitive interference measures, and the fMRI anterior cingulate cortex activation level/ connectivity during MSIT several independent 6 x 2- way ANCOVA (one per outcome measure) will be conducted (two-tailed, significance level is set at 5%) with session (number of training session) as within-subjects factor, group (fMRI-neurofeedback vs. control neurofeedback) as between-subjects factor, and age, sex and IQ as covariates.

The influence of current state of motivation on training success will be calculated by computing non-parametric Spearman's rank correlations within each individual patient.

The mental strategies used will be scored and their influence on training success will be evaluated by computing non-parametric Spearman's rank correlations within each individual patient.

Related to secondary objective (2): Investigate if the abnormal activation levels in ADHD patients play a causal role in ADHD pathophysiology.

To estimate the influence of the neurofeedback training on the symptoms a measure of change in activation level during the neurofeedback training/ the transfer blocks at the end of the training will be developed. This measure (sessions x subjects) will be correlated with the performance on the MSIT task in the subsequent session within and across the two groups (fMRI-neurofeedback and control neurofeedback).

9. Ethical Considerations

Regulation statement

The study will be conducted according to the principles of the "Declaration of Helsinki" (as amended in Tokyo, Venice and Hong Kong) and in accordance with the Guideline for Good Clinical Practice (GCP) (CPMP/ICH/135/95 - 17th July 1996). Any or all of the recommendations, requests, or documents addressed in the ICH guideline for Good Clinical Practice may be subject to, and must be available for, an audit through competent authorities (inspection). Investigational sites, facilities, and laboratories, and all data (including source data) and documentation must be available for competent authorities. The protocol of this study will be submitted to the “Centrale Commissie Mensgebonden Onderzoek" (CCMO), and the recruitment of volunteers will not commence before formal approval has been granted. The investigators must be aware of their responsibilities, as described in Chapter 4 of the ICH guideline for Good Clinical Practice and the regulatory regulations.

Benefits and risks assessment

For all ADHD subjects treatment effect is prospected. The risks of this study are estimated as very low. Potential benefit of this study is not only expected for the subjects of this study in terms of treatment response, but for all ADHD patients in terms of expanding knowledge and extending treatment opportunities.

Compensation for injury

Subjects are insured by the UMC St Radboud in Nijmegen. See for further information, *‘verzekeringsinformatie’, section E1.*

Incentives

The participants receive a gift certificate with a value of 20 euro during the evaluation.

10. Administrative Aspects and Publication

Handling and storage of data and documents

All study data will be handled confidentially. After the selection period, a code number will be given to each subject. A list of this code numbers will be stored by the principal investigator (prof. dr. J.K. Buitelaar). The code numbers will further identify the subjects and their treatments, documents, etc. The principal investigator will keep a record relating the names of all subjects that have given their informed consent, to their code numbers, to allow easy checking of data in subject files, when required. This record will also include dates of subject enrolment and completion, as well as subjects who could not be randomized for whatever reason. The investigator will retain the originals of all source documents for a period of 2 years after the report of the study has been finalized, after which all study-related documents will be archived for at least 15 years according to GCP regulations. All data and documents must be made available if requested by relevant authorities. Records must be maintained to verify the existence of each subject in the clinical trial and must contain their full names, last known addresses, telephone numbers, and other pertinent information. Data will be stored in a distinctive research status for each of the subjects.

Amendments

Amendments are changes made to the research after a favorable opinion by the accredited METC has been given. All amendments will be notified to the METC that gave a favorable opinion.

Annual progress report

The investigator will submit a summary of the progress of the trial to the accredited METC once a year. Information will be provided on the date of inclusion of the first subject, numbers of subjects included and numbers of subjects that have completed the trial, serious adverse events/ serious adverse reactions, other problems, and amendments.

End of study report

The investigator will notify the accredited METC of the end of the study within a period of 8 weeks. The end of the study is defined as the last patient ’ s last visit. In case the study is ended prematurely, the investigator will notify the accredited METC, including the reasons for the premature termination. Within one year after the end of the study, the investigator/sponsor will submit a final study report with the results of the study, including any publications/abstracts of the study, to the accredited METC.

Public disclosure and publication policy

The results of this study will be described in a PhD thesis and submitted to peer-reviewed journals.

11. References

American Psychiatric Association. (2000). *Diagnostic and Statistical Manual of Mental Disorders - 4th Edition-Text Revision*. Washington DC: American Psychiatric Press.

Amico, F., Stauber, J., Koutsouleris, N., & Frodl, T. (2011). Anterior cingulate cortex gray matter abnormalities in adults with attention deficit hyperactivity disorder: a voxel-based morphometry study. *Psychiatry Res, 191*(1), 31-35.

Antshel, K. M., Faraone, S. V., Stallone, K., Nave, A., Kaufmann, F. A., Doyle, A., et al. (2007). Is attention deficit hyperactivity disorder a valid diagnosis in the presence of high IQ? Results from the MGH Longitudinal Family Studies of ADHD. *J Child Psychol Psychiatry, 48*(7), 687-694.

Antshel, K. M., Hargrave, T. M., Simonescu, M., Kaul, P., Hendricks, K., & Faraone, S. V. (2011). Advances in understanding and treating ADHD. *BMC Med, 9*, 72.

Bandettini, P. A., Birn, R. M., & Donahue, K. M. (2000). Functional MRI: Background, methodology, limits, and implementation. In J. T. Cacioppo, L. G. Tassinary & G. G. Berntson (Eds.), *Handbook of Psychophysiology* (pp. 978-1014). Cambridge: University Press.

Biederman, J. (2004). Impact of comorbidity in adults with attention-deficit/hyperactivity disorder. *J Clin Psychiatry, 65 Suppl 3*, 3-7.

Borm, F. G., Hoogendoorn, E. H., den Heijer, M., Zielhuis, G. A. (2005). Sequential balancing: A simple method for treatment allocation in clinical trials. *Contem Clin Trials, 26*, 637-645.

Bush, G. (2011). Cingulate, frontal, and parietal cortical dysfunction in attention-deficit/hyperactivity disorder. *Biol Psychiatry, 69*(12), 1160-1167.

Bush, G., Frazier, J. A., Rauch, S. L., Seidman, L. J., Whalen, P. J., Jenike, M. A., et al. (1999). Anterior cingulate cortex dysfunction in attention-deficit/hyperactivity disorder revealed by fMRI and the Counting Stroop. *Biol Psychiatry, 45*(12), 1542-1552.

Bush, G., & Shin, L. M. (2006). The Multi-Source Interference Task: an fMRI task that reliably activates the cingulo-frontal-parietal cognitive/attention network. *Nat Protoc, 1*(1), 308-313.

Bush, G., Spencer, T. J., Holmes, J., Shin, L. M., Valera, E. M., Seidman, L. J., et al. (2008). Functional magnetic resonance imaging of methylphenidate and placebo in attention-deficit/hyperactivity disorder during the multi-source interference task. *Arch Gen Psychiatry, 65*(1), 102-114.

Castellanos, F. X., Margulies, D. S., Kelly, C., Uddin, L. Q., Ghaffari, M., Kirsch, A., et al. (2008). Cingulate-precuneus interactions: a new locus of dysfunction in adult attention-deficit/hyperactivity disorder. *Biol Psychiatry, 63*(3), 332-337.

Cohen, J. (1988). *Statistical power analysis for the behavioural sciences* (2 ed.). Hillsdale, N.J. : Erlbaum.

Cubillo, A., Halari, R., Ecker, C., Giampietro, V., Taylor, E., & Rubia, K. (2010). Reduced activation and inter-regional functional connectivity of fronto-striatal networks in adults with childhood Attention-Deficit Hyperactivity Disorder (ADHD) and persisting symptoms during tasks of motor inhibition and cognitive switching. *J Psychiatr Res, 44*(10), 629-639.

De Sonneville, L. (1999). Amsterdam Neuropsychological Tasks: a computer-aided assessment program. In B. d. Brinker, P. Beek, A. Brand, S. Maarse & L. Mulder (Eds.), *Cognitive ergonomics, clinical assessment and computer-assisted learning: Computers in Psychology* (Vol. 6, pp. 187-203). The Netherlands: Lisse, Swets & Zeitlinger.

deCharms, R. C., Maeda, F., Glover, G. H., Ludlow, D., Pauly, J. M., Soneji, D., et al. (2005). Control over brain activation and pain learned by using real-time functional MRI. *Proc Natl Acad Sci U S A, 102*(51), 18626-18631.

Dickstein, S. G., Bannon, K., Castellanos, F. X., & Milham, M. P. (2006). The neural correlates of attention deficit hyperactivity disorder: an ALE meta-analysis. *J Child Psychol Psychiatry, 47*(10), 1051-1062.

Faraone, S. V., & Glatt, S. J. (2010). A comparison of the efficacy of medications for adult attention-deficit/hyperactivity disorder using meta-analysis of effect sizes. *J Clin Psychiatry, 71*(6), 754-763.

Fox, D. J., Tharp, D. F., & Fox, L. C. (2005). Neurofeedback: an alternative and efficacious treatment for Attention Deficit Hyperactivity Disorder. *Appl Psychophysiol Biofeedback, 30*(4), 365-373.

Haller, S., Birbaumer, N., & Veit, R. (2010). Real-time fMRI feedback training may improve chronic tinnitus. *Eur Radiol, 20*(3), 696-703.

Hamilton, J. P., Glover, G. H., Hsu, J. J., Johnson, R. F., & Gotlib, I. H. (2011). Modulation of subgenual anterior cingulate cortex activity with real-time neurofeedback. *Hum Brain Mapp, 32*(1), 22-31.

Hammer, E. M., Halder, S., Blankertz, B., Sannelli, C., Dickhaus, T., Kleih, S., et al. (2012). Psychological predictors of SMR-BCI performance. *Biol Psychol, 89*(1), 80-86.

Hawkinson, J. E., Ross, A. J., Parthasarathy, S., Scott, D. J., Laramee, E. A., Posecion, L. J., et al. (2011). Quantification of Adverse Events Associated with Functional MRI Scanning and with Real-Time fMRI-Based Training. *Int J Behav Med*.

Heinrich, H., Gevensleben, H., & Strehl, U. (2007). Annotation: neurofeedback - train your brain to train behaviour. *J Child Psychol Psychiatry, 48*(1), 3-16.

Hirshberg, L. M. (2007). Place of electroencephalograpic biofeedback for attention-deficit/hyperactivity disorder. *Expert Rev Neurother, 7*(4), 315-319.

Ivers, N. M., Halperin, I. J., Barnsley, J., Grimshaw, J. M., Shah, B. R., Tu, K., et al. (2012). Allocation techniques for balance at baseline in cluster randomized trials: a methodological review. *Trials, 13*(1), 120.

Kirby, A. J., Galai, N., & Munoz, A. (1994). Sample size estimation using repeated measurements on biomarkers as outcomes. *Control Clin Trials, 15*(3), 165-172.

Konrad, A., Dielentheis, T. F., El Masri, D., Bayerl, M., Fehr, C., Gesierich, T., et al. (2010). Disturbed structural connectivity is related to inattention and impulsivity in adult attention deficit hyperactivity disorder. *Eur J Neurosci, 31*(5), 912-919.

Kooij, J. J., Buitelaar, J. K., van den Oord, E. J., Furer, J. W., Rijnders, C. A., & Hodiamont, P. P. (2005). Internal and external validity of attention-deficit hyperactivity disorder in a population-based sample of adults. *Psychol Med, 35*(6), 817-827.

Loo, S. K., & Barkley, R. A. (2005). Clinical utility of EEG in attention deficit hyperactivity disorder. *Appl Neuropsychol, 12*(2), 64-76.

Makris, N., Biederman, J., Valera, E. M., Bush, G., Kaiser, J., Kennedy, D. N., et al. (2007). Cortical thinning of the attention and executive function networks in adults with attention-deficit/hyperactivity disorder. *Cereb Cortex, 17*(6), 1364-1375.

Nijboer, F., Birbaumer, N., & Kubler, A. (2010). The influence of psychological state and motivation on brain-computer interface performance in patients with amyotrophic lateral sclerosis - a longitudinal study. *Front Neurosci, 4*.

Rheinberg, F., Vollmeyer, R., & Burns, B. (2001). FAM: Eine Fragebogen zur Erfassung Aktueller Motivation in Lern- und Leistungssituationen. *Diagnostica, 47*, 57-66.

Rossiter, T. (2004). The effectiveness of neurofeedback and stimulant drugs in treating AD/HD: Part I. Review of methodological issues. *Appl Psychophysiol Biofeedback, 29*(2), 95-112.

Ruiz, S., Lee, S., Soekadar, S. R., Caria, A., Veit, R., Kircher, T., et al. (2011). Acquired self-control of insula cortex modulates emotion recognition and brain network connectivity in schizophrenia. *Hum Brain Mapp*, [Epub ahead of print].

Santosh, P. J., Sattar, S., & Canagaratnam, M. (2011). Efficacy and tolerability of pharmacotherapies for attention-deficit hyperactivity disorder in adults. *CNS Drugs, 25*(9), 737-763.

Schenck, J. F. (2000). Safety of strong, static magnetic fields. *J Magn Reson Imaging, 12*(1), 2-19.

Schneider, M. F., Krick, C. M., Retz, W., Hengesch, G., Retz-Junginger, P., Reith, W., et al. (2010). Impairment of fronto-striatal and parietal cerebral networks correlates with attention deficit hyperactivity disorder (ADHD) psychopathology in adults - a functional magnetic resonance imaging (fMRI) study. *Psychiatry Res, 183*(1), 75-84.

Scott, N. W., McPherson, G. C., Ramsay, C. R., & Campbell, M. K. (2002). The method of minimization for allocation to clinical trials: a review. *Control Clin Trials, 23*(6), 662-674.

Secnik, K., Swensen, A., & Lage, M. J. (2005). Comorbidities and costs of adult patients diagnosed with attention-deficit hyperactivity disorder. *Pharmacoeconomics, 23*(1), 93-102.

Seidman, L. J., Biederman, J., Liang, L., Valera, E. M., Monuteaux, M. C., Brown, A., et al. (2011). Gray matter alterations in adults with attention-deficit/hyperactivity disorder identified by voxel based morphometry. *Biol Psychiatry, 69*(9), 857-866.

Seidman, L. J., Valera, E. M., Makris, N., Monuteaux, M. C., Boriel, D. L., Kelkar, K., et al. (2006). Dorsolateral prefrontal and anterior cingulate cortex volumetric abnormalities in adults with attention-deficit/hyperactivity disorder identified by magnetic resonance imaging. *Biol Psychiatry, 60*(10), 1071-1080.

Sitaram, R., Veit, R., Stevens, B., Caria, A., Gerloff, C., Birbaumer, N., et al. (2011). Acquired Control of Ventral Premotor Cortex Activity by Feedback Training: An Exploratory Real-Time fMRI and TMS Study. *Neurorehabil Neural Repair*, [Epub ahead of print].

Sorger, B. (2010). *When the brain speaks for itself. Exploiting hemodynamic brain signals for motor-independent communication.* Maastricht University, Maastricht.

Spencer, T. J., Biederman, J., & Mick, E. (2007). Attention-deficit/hyperactivity disorder: diagnosis, lifespan, comorbidities, and neurobiology. *J Pediatr Psychol, 32*(6), 631-642.

Sterman, M. B. (1996). Physiological origins and functional correlates of EEG rhythmic activities: implications for self-regulation. *Biofeedback Self Regul, 21*(1), 3-33.

Subramanian, L., Hindle, J. V., Johnston, S., Roberts, M. V., Husain, M., Goebel, R., et al. Real-time functional magnetic resonance imaging neurofeedback for treatment of Parkinson's disease. *J Neurosci, 31*(45), 16309-16317.

Subramanian, L., Hindle, J. V., Johnston, S., Roberts, M. V., Husain, M., Goebel, R., et al. (2011). Real-time functional magnetic resonance imaging neurofeedback for treatment of Parkinson's disease. *J Neurosci, 31*(45), 16309-16317.

Uterwijk, J. (2005). *Nederlandstalige bewerking van de Wechsler Adult Intelligence Scale, WAIS-III van D. Wechsler (1997)*. The Netherlands: Lisse: Swets & Zeitlinger.

Veit, R. (2009). Echtzeit-fMRT und Therapie. In *Neurobiologie forensisch relevanter Stoerungen*. Stuttgart: Kohlhammer.

Weiskopf, N. (in press). Real-time fMRI and its application to neurofeedback. *Neuroimage*.

Weiskopf, N., Sitaram, R., Josephs, O., Veit, R., Scharnowski, F., Goebel, R., et al. (2007). Real-time functional magnetic resonance imaging: methods and applications. *Magn Reson Imaging, 25*(6), 989-1003.

Weiskopf, N., Veit, R., Erb, M., Mathiak, K., Grodd, W., Goebel, R., et al. (2003). Physiological self-regulation of regional brain activity using real-time functional magnetic resonance imaging (fMRI): methodology and exemplary data. *Neuroimage, 19*(3), 577-586.

Willcutt, E. G., Doyle, A. E., Nigg, J. T., Faraone, S. V., & Pennington, B. F. (2005). Validity of the executive function theory of attention-deficit/hyperactivity disorder: a meta-analytic review. *Biol Psychiatry, 57*(11), 1336-1346.

Woods, D. L., Kishiyama, M. M., Yund, E. W., Herron, T. J., Edwards, B., Poliva, O., et al. (2011). Improving digit span assessment of short-term verbal memory. *J Clin Exp Neuropsychol*, 1-11.
