# Supplementary material for: fMRI Neurofeedback Training for Increasing Anterior Cingulate Cortex Activation in Adult Attention Deficit Hyperactivity Disorder. An Exploratory Randomized, Single-Blinded Study
Source: PLoS One. 2017 Jan 26;12(1):e0170795. doi: 10.1371/journal.pone.0170795 (PMC5270326; doi:10.1371/journal.pone.0170795)
Supplement: S1 Table — The x, y and z coordinates in Talairach space of the individual dACC target regions are shown per subject and session. SD = standard deviation. (PDF) [file pone.0170795.s009.pdf]

Neurofeedback group

	session 1			session 2			session 3			session 4			session 1-4		
	x	y	z	x	y	z	x	y	z	x	y	z	x	y	z
S11	6	21	41	-1	16	41	-7	15	38	-2	17	41	-1	17	40
S12	9	12	42	8	17	39	8	10	41	4	9	41	7	12	41
S13	3	14	40	-2	12	41	-3	12	44				-1	13	42
S14	-3	24	43	-3	23	44	-4	1	49	-4	2	46	-4	13	46
S15	4	15	41	-3	23	38	1	16	41	-1	18	39	0	18	40
S16	3	21	38	3	20	35	6	23	34	8	22	34	5	22	35
S17	1	6	31	10	6	34	3	19	34	2	12	34	4	11	33
**mean**	**3**	**16**	**39**	**2**	**17**	**39**	**1**	**14**	**40**	**1**	**13**	**39**	**2**	**15**	**39**
SD	4	6	4	5	6	4	6	7	5	4	7	5	4	4	4

Control group

	session 1			session 2			session 3			session 4			session 1-4		
	x	y	z	x	y	z	x	y	z	x	y	z	x	y	z
S21	1	18	41	-1	13	35	4	14	39	3	11	44	2	14	40
S22	8	22	31	5	8	28	5	27	39				6	19	33
S23	5	14	40	5	10	42	4	21	37	7	13	41	5	15	40
S24	-7	25	30	-3	22	38	-8	11	47	-4	23	30	-6	20	36
S25	-6	1	29	-5	5	34	-4	7	35	-7	10	35	-6	6	33
S26	1	26	35	5	19	32	4	29	32	11	11	38	5	21	34
**mean**	**0**	**18**	**34**	**1**	**13**	**35**	**1**	**18**	**38**	**2**	**14**	**38**	**1**	**16**	**36**
SD	6	9	5	5	7	5	5	9	5	7	5	5	5	6	3
