# Supplementary material for: fMRI Neurofeedback Training for Increasing Anterior Cingulate Cortex Activation in Adult Attention Deficit Hyperactivity Disorder. An Exploratory Randomized, Single-Blinded Study
Source: PLoS One. 2017 Jan 26;12(1):e0170795. doi: 10.1371/journal.pone.0170795 (PMC5270326; doi:10.1371/journal.pone.0170795)
Supplement: S2 Table — Significant effects (p ≤ 0.05) are printed bold and marked with an asterisk. WAIS = Wechsler Adult Intelligence Scale, ADHD = Attention Deficit Hyperactivity Disorder, VC = Vocabulary, BD = Block Design, MSIT = Multi Source Interference task, SA-DOTS = Sustained Attention DOTS task, SART = Sustained Attention to Response Task, WM = Working memory, DS = Digit Span, LNS = Letter Number Sequencing. (PDF) [file pone.0170795.s010.pdf]

**Predictors** (training session/pre-test)	**Controls (n=6)** correlation (p-value)	**Neurofeedback (n=7)** correlation (p-value)
**Predicting *general task performance* (mean activation level across session)**		
Motion (mean displacement)	0.03 (p = 0.97)	**-0.79 (p = 0.03) ***
Motivation (QCM score)	0.03 (p = 0.95)	0.26 (p = 0.58)
IQ (WAIS VC/ BD)	0.65 (p = 0.16)	-0.11 (p = 0.82)
ADHD attention (DSM-IV score)	-0.22 (p = 0.68)	-0.69 (p = 0.09)
ADHD impulsivity/hyperactivity (DSM-IV score)	-0.57 (p = 0.23)	0.17 (p = 0.72)
Interference (MSIT, interference delay ms)	0.56 (p = 0.24)	-0.27 (p = 0.56)
Vigilance (SA-DOTS, false alarms)	-0.78 (p = 0.06)	0.63 (p = 0.13)
Response inhibition (SA-DOTS, % missed)	-0.60 (p = 0.21)	-0.29 (p = 0.53)
Response inhibition (SART, % false alarms)	-0.72 (p = 0.11)	-0.75 (p = 0.05)
Visual WM accuracy (2-back, % accuracy)	-0.24 (p = 0.64)	0.43 (p = 0.34)
Verbal WM (WAIS DS, IQ score)	0.23 (p = 0.66)	-0.03 (p = 0.95)
Verbal WM (WAIS LNS, IQ score)	0.39 (p = 0.44)	0.03 (p = 0.96)
**Predicting *improvement over sessions* (increase in activation level over sessions)**		
Motion (mean displacement)	-0.27 (p = 0.60)	-0.63 (p = 0.13)
Motivation (QCM score)	-0.28 (p = 0.59)	0.71 (p = 0.08)
IQ (WAIS VC/ BD)	0.67 (p = 0.13)	0.32 (p = 0.48)
ADHD attention (DSM-IV score)	-0.39 (p = 0.45)	-0.21 (p = 0.65)
ADHD impulsivity/hyperactivity (DSM-IV score)	0.03 (p = 0.96)	-0.37 (p = 0.42)
Interference (MSIT, interference delay ms)	0.65 (p = 0.16)	-0.41 (p = 0.37)
Vigilance (SA-DOTS, false alarms)	-0.47 (p = 0.35)	0.52 (p = 0.23)
Response inhibition (SA-DOTS, % missed)	0.24 (p = 0.65)	-0.36 (p = 0.42)
Response inhibition (SART, % false alarms)	-0.33 (p = 0.52)	**-0.80 (p = 0.03) ***
Visual WM accuracy (2-back, % accuracy)	0.40 (p = 0.43)	**0.86 (p = 0.01) ***
Verbal WM (WAIS DS, IQ score)	-0.34 (p = 0.51)	0.28 (p = 0.54)
Verbal WM (WAIS LNS, IQ score)	-0.24 (p = 0.65)	0.46 (p = 0.30)

Predicting *improvement in differential modulation* (increase in differential activation)		
Motion (mean displacement)	-0.31 (p = 0.54)	-0.65 (p = 0.12)
Motivation (QCM score)	-0.55 (p = 0.26)	0.21 (p = 0.65)
IQ (WAIS VC/ BD)	0.72 (p = 0.10)	0.10 (p = 0.82)
ADHD attention (DSM-IV score)	-0.67 (p = 0.15)	-0.20 (p = 0.67)
ADHD impulsivity/hyperactivity (DSM-IV score)	-0.05 (p = 0.93)	0.09 (p = 0.85)
Interference (MSIT, interference delay ms)	-0.29 (p = 0.57)	-0.19 (p = 0.68)
Vigilance (SA-DOTS, false alarms)	-0.08 (p = 0.88)	0.003 (p = 0.99)
Response inhibition (SA-DOTS, % missed)	0.05 (p = 0.93)	**-0.91 (p = 0.005) ***
Response inhibition (SART, % false alarms)	-0.10 (p = 0.85)	-0.47 (p = 0.28)
Visual WM accuracy (2-back, % accuracy)	0.68 (p = 0.13)	0.62 (p = 0.14)
Verbal WM (WAIS DS, IQ score)	0.58 (p = 0.23)	**0.81 (p = 0.03) ***
Verbal WM (WAIS LNS, IQ score)	0.16 (p = 0.77)	**0.80 (p = 0.03) ***
